# HMGB2–RAD21 Axis Promotes Fibro/Adipogenic Progenitor Proliferation and Regulates Fat Infiltration

**DOI:** 10.1002/advs.202514363

**Published:** 2026-01-12

**Authors:** Xian Tong, Ziyun Liang, Tianqi Duo, Liping Pan, Qi Zhu, Jiete Liang, Xianyao Luo, Qingcai Feng, Rong Xu, Yihao Liu, Xu Chen, Luxi Chen, Xiaohong Liu, Yaosheng Chen, Delin Mo

**Affiliations:** ^1^ State Key Laboratory of Biocontrol School of Life Sciences Sun Yat‐Sen University Guangzhou Guangdong China

**Keywords:** adipogenic differentiation, fibro/adipogenic progenitors, HMGB2, intermuscular fat, single‐cell RNA sequencing

## Abstract

Intermuscular fat (IMF) infiltration is not only associated with myopathies and insulin resistance, but also serves as a key determinant of meat quality in the livestock industry. However, the molecular and cellular mechanisms influencing the intermuscular adipocyte abundance remain poorly understood. Based on porcine samples, we confirmed that the differentiation of intermuscular preadipocytes begins after birth, which prompted us to focus on the changes in the number of fibro/adipogenic progenitors (FAPs) during the embryonic stage. Using single‐cell sequencing (ScRNA‐seq) analysis of pig embryonic muscle, we constructed the first developmental atlas of embryonic FAPs and identified a distinct HMGB2^+^ subpopulation (FAPs^HMGB2+^) as a key determinant of FAP pool size. When HMGB2 is knocked out, both heterozygous and homozygous mice exhibit a remarkable reduction in the number of FAPs and impaired adipogenic potential. Correspondingly, the FAPs^HMGB2+^ were also found during muscle regeneration in mice. Unlike targeting C/EBPβ in vitro, *HMGB2* governs FAP proliferation in vivo through targeting *RAD21*, a gene involved in DNA replication. Collectively, these findings provide novel insights into analyzing differences in IMF content and highlight potential targets for enhancing pork quality and mitigating pathological fat infiltration in skeletal muscles.

## Graphical abstract

This study constructed the first developmental atlas of embryonic skeletal muscle fibro/adipogenic progenitors (FAPs) and identified an HMGB2^+^ FAPs subpopulation that regulates FAP pool size and muscle homeostasis. HMGB2 directly targets the RAD21 promoter, and its knockout significantly reduces FAP numbers, thereby lowering the potential for intermuscular adipocyte deposition. These findings provide novel insights into the mechanisms of fat infiltration.

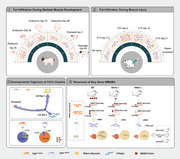

## 1 Introduction

Skeletal muscle, which comprises approximately 35%–45% of total body mass and contributes around 30% to the basal metabolic rate [1], is one of the largest and most metabolically active organs in the body. Intermuscular fat (IMF), a specialized adipose depot located within skeletal muscle, is closely associated with various myopathies [2]. In livestock, IMF, often referred to as marbling, serves as a key determinant of meat quality, directly influencing the meat's visual appeal, juiciness, flavor, and overall palatability [3]. Thus, elucidating the biological mechanisms that drive IMF accumulation is crucial for developing innovative approaches to enhance meat quality.

Fibro/adipogenic progenitors (FAPs), a type of mesenchymal stem cell located between muscle fibers, are the primary source of adipocytes in skeletal muscle [4, 5]. FAPs play a critical role in muscle development and growth [6], both in maintaining tissue structure and function [7, 8] and in regulating fat deposition [9, 10, 11]. When the quantity or differentiation of FAPs changes, the formation and distribution of IMF also change, directly affecting meat quality traits [12, 13]. Our team previously demonstrated that in the skeletal muscle of newborn piglets from breeds with large differences in IMF content, the number of FAPs varied by up to four times [14]. Therefore, modulating FAP activity to regulate adipocyte formation while supporting myogenesis is considered a feasible strategy for improving meat quality.

FAPs exhibit functional heterogeneity, characterized by diverse differentiation capacities and the ability to adopt distinct cell fates in response to environmental cues [15, 16, 17, 18]. Many studies have shown that FAPs can develop into either adipocytes or fibroblasts that produce type I collagen [18]. Intermuscular fat, together with subcutaneous and visceral fat, belongs to white adipose tissue [14]. In mouse subcutaneous and visceral white fat, PDGFRα^+^ cells can be divided into two groups based on CD9 expression levels: CD9 ^low^/PDGFRα^+^ cells can become adipocytes and are considered preadipocytes, while CD9 ^high^/PDGFRα^+^ cells lack adipogenic ability and are often linked to fibrosis [19, 20]. Our team further demonstrated that adipocytes in skeletal muscle mainly come from CD9‐negative FAPs, which have strong fat‐forming potential [14]. Furthermore, the regulation of FAPs function is mediated by multiple signaling pathways and key genes, including Notch signaling [16], Hedgehog (Hh) signaling [21], AMPK Pathway [22], vitamin D receptor [23], and TGF‐β signaling [24], which influence their adipogenic potential. However, the mechanisms that drive IMF accumulation remain inadequately understood. Addressing these research gaps is essential for advancing our understanding of the role of FAPs in maintaining tissue homeostasis, improving meat quality, and elucidating their contributions to the progression of muscle‐related diseases.

Intermuscular adipocytes are present in healthy adults and tend to accumulate progressively with age [21, 25]. However, the timing of their differentiation in mammalian skeletal muscle remains unknown. In contrast, adipocytes are rarely found in the skeletal muscle of healthy adult mice. Research on fat infiltration in mouse skeletal muscle mainly focuses on disease models such as Duchenne muscular dystrophy (DMD), type 2 diabetes, or aging [26], suggesting that mice may not be an ideal model for studying the timing of intermuscular adipocyte differentiation. Pigs exhibit greater genetic and physiological similarities to humans compared to mice, particularly in terms of genome composition and the size of key organs, rendering them a valuable model for biomedical research [27]. This physiological resemblance has contributed to the growing use of pigs in studies of metabolic regulation and disease [28]. However, most ScRNA‐seq studies of intermuscular fat in pigs have focused on the adult stage [12, 13, 28], overlooking the full developmental trajectory of intermuscular adipogenesis, making it fundamentally impossible to determine the factors that govern the number of intermuscular adipocytes.

**FIGURE 1:**
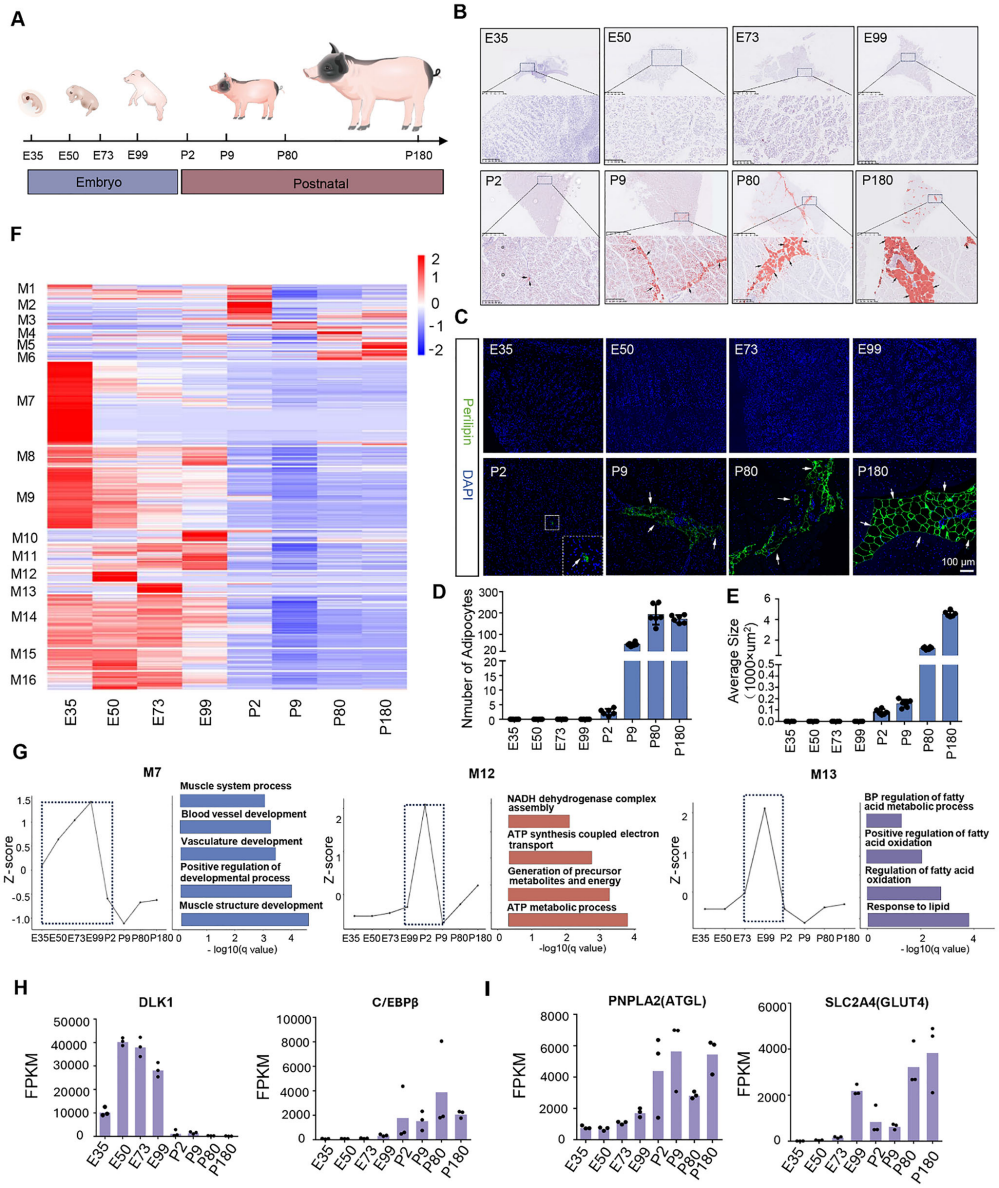
Temporal Dynamics of Intermuscular Adipocyte Differentiation in Skeletal Muscle. (A) LD samples were collected from Guangdong small‐eared spotted pigs at eight developmental time points: embryonic days (E) and postnatal days (P). (B) Oil Red O staining of LD samples from various developmental stages. (C) Perilipin immunofluorescence staining of LD samples across different developmental stages. (D) Quantification of the number of intermuscular adipocytes. (E) Quantification of the size of intermuscular adipocytes. (F) Heatmap displaying the expression patterns of genes in the 16 co‐expression modules (Three biological replicates were included at each developmental stage). (G) Expression Changes and GO Enrichment Analysis of Modules 7, 12, and 13. The left panel shows the median Z score of genes in each module across skeletal muscle development, while the right panel lists the top five or four typical GO biological process terms associated with each module. (H, I) Changes in FPKM levels of glucose metabolism‐related gene (GLUT4) and adipogenesis genes (DLK1, C/EBβ, and ATGL) during skeletal muscle development.

HMGB2 is a non‐histone protein belonging to the HMGB (High‐Mobility Group) superfamily and contains a conserved HMGB domain [29, 30]. HMG proteins, first identified in the mid‐1970s due to their high electrophoretic mobility, are divided into three families: HMGA, HMGN, and HMGB [31]. HMGB proteins, including HMGB1–4, interact with DNA through their HMGB domains [31]. Initially characterized as structural chromatin components, HMGB1 and HMGB2 are now known to possess a broader functional repertoire, including key roles in chromatin remodeling, transcription regulation, and DNA repair. In particular, HMGB2 expression is closely associated with critical cellular processes such as differentiation, senescence, and the maintenance of stem cell potency [32]. Pertinently, Lee et al. implicated HMGB2 in the regulation of fat infiltration during muscle repair. They demonstrated that HMGB2 is highly expressed in undifferentiated mesenchymal stem cells (MSCs) and co‐localizes with PDGFRα, and that the loss of HMGB2 markedly impairs adipogenic differentiation in MSCs, accompanied by reduced PDGFRα expression [33]. Nonetheless, the precise molecular mechanisms underlying this function remain unknown. A previous study from our team has shown that HMGB2 can modulate mitotic clonal expansion (MCE) of 3T3‐L1 by binding to the promoter of C/EBPβ in vitro [34]. Moreover, C/EBPβ is barely expressed in embryonic skeletal muscle (Figure 1F), further suggesting that HMGB2 does not regulate embryonic FAP proliferation by targeting C/EBPβ.

In this study, longissimus dorsi samples separated from Guangdong small‐eared spotted pig (GDSS) at embryonic, juvenile, and adult stages (*N* = 3 per stage) were analyzed using histological staining and bulk RNA sequencing. Intermuscular adipocytes were detectable as early as the neonatal stage (P2). Using ScRNA‐seq, we constructed the first developmental atlas of embryonic FAPs in porcine skeletal muscle and identified a distinct subpopulation of FAP subpopulation characterized by high expression of HMGB2 (FAPs^HMGB2+^). This subpopulation was found to govern FAPs pool size during embryonic periods and affect intermuscular adipocyte abundance postnatally. Furthermore, the FAPs^HMGB2+^ were also found during muscle regeneration in mice. HMGB2 was identified as a key gene regulating FAPs proliferation by targeting RAD21 in mice. These findings offer new insights into the mechanisms underlying intermuscular adipocyte development prenatally and highlight the potential target for enhancing pork quality and mitigating fat infiltration in skeletal muscle.

## 2 Results

### 2.1 Intermuscular Adipocyte Differentiation Occurs Postnatally

The differentiation of intermuscular adipocytes was evaluated by Oil Red O staining and Perilipin immunofluorescence staining using longissimus dorsi (LD) muscle samples from Guangdong small‐eared spotted pigs (GDSS), a pig breed characterized by high IMF content. Samples were collected at multiple developmental stages, including embryonic stages (E35, E50, E73, E99) and postnatal stages (P2, P9, P80, P180) (Figure 1A). Adipocyte morphology became notably visible as early as postnatal day 2 (P2, samples harvested at 36 h after birth), with intermuscular fat content progressively increasing at P9, P80, and P180 (Figure 1B,C).

To investigate why intermuscular adipocyte differentiation occurs only after birth, we analyzed the transcriptome of muscle tissue to identify potential factors related to changes in the microenvironment surrounding intermuscular adipocytes before and after birth. To this end, bulk RNA sequencing of LD muscle samples across all developmental stages, with three biological replicates per stage, was performed and generated approximately 108.99 million high‐quality reads (Tables S5 and S6). Genes were clustered into 16 co‐expression modules using the K‐means algorithm (Figure 1D). GO enrichment analysis revealed spatio‐temporal expression patterns linked to distinct biological functions, with particular attention to modules related to muscle development, and notably, lipid and energy metabolism, both of which are key drivers of adipocyte differentiation (Figure 1E; Figure S1A). Module 7 (M7), associated with muscle development, exhibited higher expression during the embryonic period (Figure 1E). Furthermore, there was a surge in lipid metabolism and energy metabolism in skeletal muscle during the late embryonic stage (M12, M13, M15, and M16), potentially contributing to the differentiation of intermuscular adipocytes postnatally (Figure 1E; Figure S1A).

Significant differences were observed in the expression levels of key regulatory genes associated with glucose metabolism (GLUT4), adipogenesis (DLK1, C/EBPβ, ATGL), and muscle development (PAX7, MyoD, MYF5, MyoG) before and after birth (Figure 1F; Figure S1B). GLUT4 and ATGL, belonging to Modules 15 and 16, respectively, began expressing in the late embryonic stage, while DLK1 from Module 2, a precursor adipocyte marker, exhibited high expression during the embryonic period but sharply decreased at P2. Conversely, C/EBPβ from Module 15, a key regulatory gene for adipogenesis, exhibited lower expression during the embryonic period but showed a remarkable increase at P2, suggesting that intermuscular adipocyte differentiation predominantly occurs postnatally (Figure 1F; Figure S1A). Additionally, genes related to muscle development were highly expressed during embryogenesis, consistent with the expression profile of Module 7 (Figure S1B).

### 2.2 The Developmental Trajectory of FAPs Subpopulations

**FIGURE 2:**
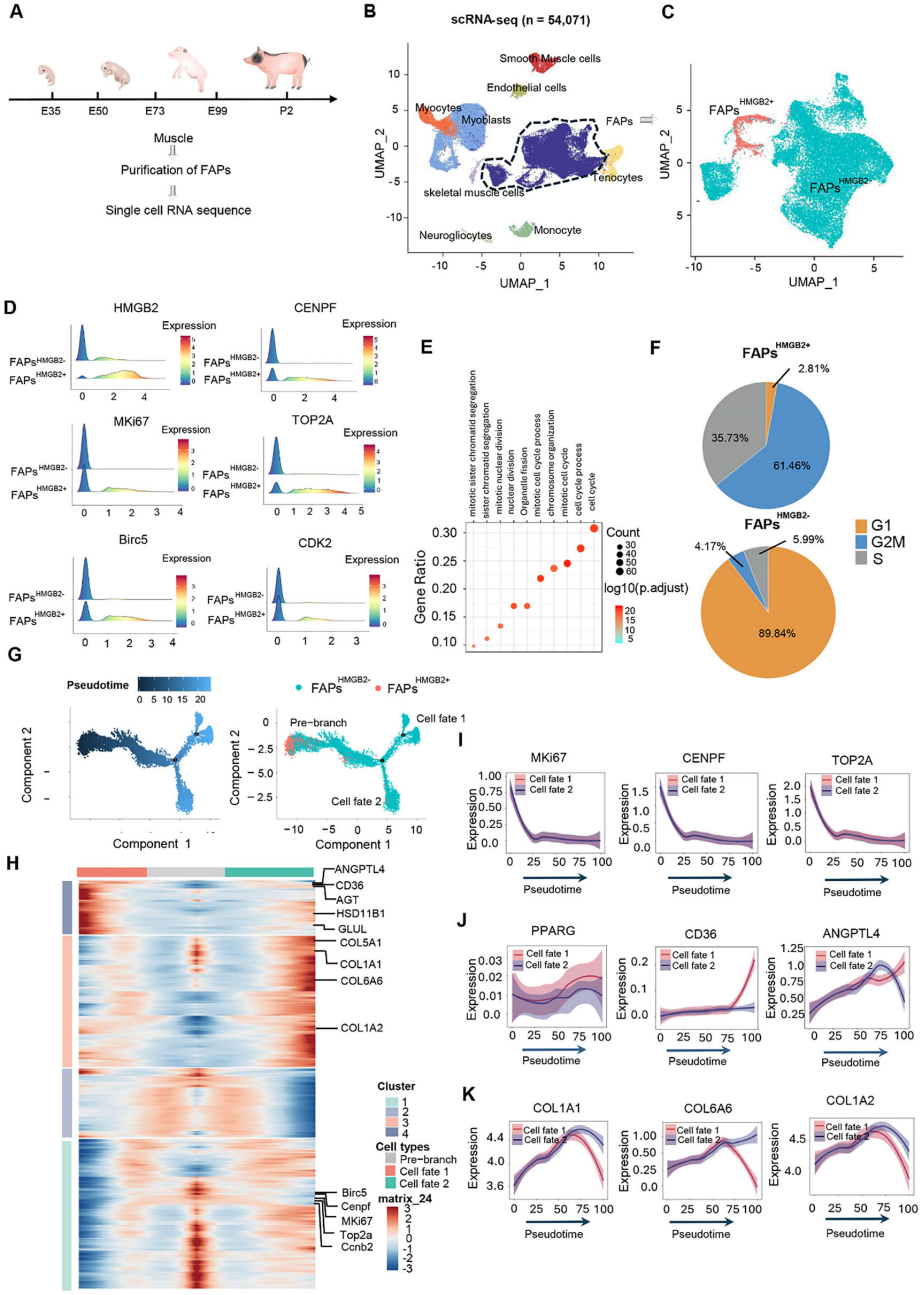
The Developmental Trajectory of FAPs Subpopulations During the Development of Embryonic Skeletal Muscle. (A) Schematic representation of porcine muscle preparation, single‐cell isolation, and ScRNA‐seq procedure. (B) Graph‐based clustering of the isolated single cells reveals distinct clusters representing different cell populations. (C) Graph‐based clustering analysis reveals two distinct subclusters in the FAPs population: FAPs^HMGB2−^ and FAPs^HMGB2+^. (D) Expression of Representative Genes in the FAPs^HMGB2+^ Cluster. (E) GO enrichment analysis of highly expressed genes in FAPs^HMGB2+^ Cluster. (F) Proportion of cell cycle in different FAPs subpopulations. (G) Pseudotime analysis of FAPs performed by Monocle 2, revealing three different cell states: Pre‐branch, Cell fate 1, and Cell fate 2. The distributions of cell states are presented along the pseudotime trajectory, with each dot representing a cell. (H) Heatmap illustrating the expression alterations of 3,941 top differentially expressed genes (DEGs) arranged in pseudotemporal order, categorized into four main clusters exhibiting distinct patterns. (I) Dynamic expression of proliferation‐associated genes (e.g., Ki67, CENPF, and Top2A). (J, K) Dynamic expression of genes related to adipogenesis and collagenation.

To better investigate the developmental trajectory of FAPs and quantify their abundance before and after intermuscular adipocyte differentiation, we collected LD muscle samples during embryonic stages E35, E50, E73, E99, and postnatal stage P2 (Figure 2A). Due to the lack of specific flow cytometry antibodies against porcine FAPs, the preplate method was used to enrich FAPs as much as possible (Figure S1C). The adherent cells were then digested and used for ScRNA‐seq. Consequently, 54 071 cells were obtained after quality control (Figure 2B). Unsupervised clustering and UMAP (Uniform Manifold Approximation and Projection) visualization identified nine distinct clusters (Figure 2B; Figure S2A–C). SNN clustering divided FAPs into two subgroups, and HMGB2 was selected as a classifier due to its marked differential expression between the clusters. (Figure 2C; Table S7). GO enrichment analysis of genes in the FAPs^HMGB2+^ cluster revealed significant enrichment in biological processes such as “Cell cycle,” “Cell cycle process,” and “Chromosome organization” (Figure 2E). Proliferation‐related genes such as MKi67, CENPF, TOP2A, Birc5, and CDK2 were specifically highly expressed in the FAPs^HMGB2+^ cluster (Figure 2D). Cell cycle analysis demonstrated that approximately 61.46% of FAPs^HMGB2+^ were in the G2/M phase, 35.73% in the S phase, and 2.81% in the G1 phase (Figure 2F). Furthermore, multiplex immunostaining of HMGB2, PDGFRα, and Ki67 further corroborated that FAPs^HMGB2+^ exhibit a high proliferative capacity (Figure S4A–C).

Pseudotime trajectory analysis of FAPs subpopulations was performed using Monocle2. Three distinct cell states were identified: Pre‐branch, Cell fate 1, and Cell fate 2, with their distribution mapped along the pseudotime trajectory (Figure 2G). Genes expressed in the Pre‐branch state were primarily enriched in cell cycle‐related terms, including “cell cycle process” and “mitotic cell cycle” (Figure S2E). As the trajectory progressed, the Pre‐branch state diverged into two distinct branches at pivot point 1. Gene expression in Cell Fate 2 was predominantly associated with collagen production (Figure S2E), while expression in Cell Fate 1 was primarily linked to adipogenesis (Figure S2E). FAPs^HMGB2+^ were predominantly located at the initial end of the Pre‐branch state within the pseudotime trajectory (Figure 2G).

To elucidate the dynamic regulation of gene expression during FAPs development, the expression patterns of 3,941 differentially expressed genes (DEGs) were analyzed across the three identified FAPs branches. Their expression patterns were grouped into four major transcriptional clusters (Figure 2H). Gene cluster 1, primarily associated with proliferation (MKi67, CENPF, BIRC5, Top2A, and Ccnb2), exhibited decreased expression as the Pre‐branch state transitioned to Cell fate 1 and Cell fate 2 (Figure 2H,I). In contrast, gene cluster 3, which includes collagen‐related genes (e.g., COL1A2, COL6A6, COL1A1, and COL5A1), showed an upward trend as the trajectory moved from the Pre‐branch state to Cell fate 2 (Figure 2H,K). Additionally, gene cluster 4, related to adipogenic differentiation (e.g., ANGPTL4, CD36, AGT, HSD11B1, and GLUL), displayed increased expression as the trajectory advanced from the Pre‐branch state to Cell fate 1 (Figure 2H,J). In conclusion, this study analyzed the developmental trajectory of FAPs subpopulations during embryonic development and identified a specific proliferating FAP subgroup, FAPs^HMGB2+^.

### 2.3 FAPs^HMGB2+^ are Predominantly Present During the Development of Embryonic Skeletal Muscle

**FIGURE 3:**
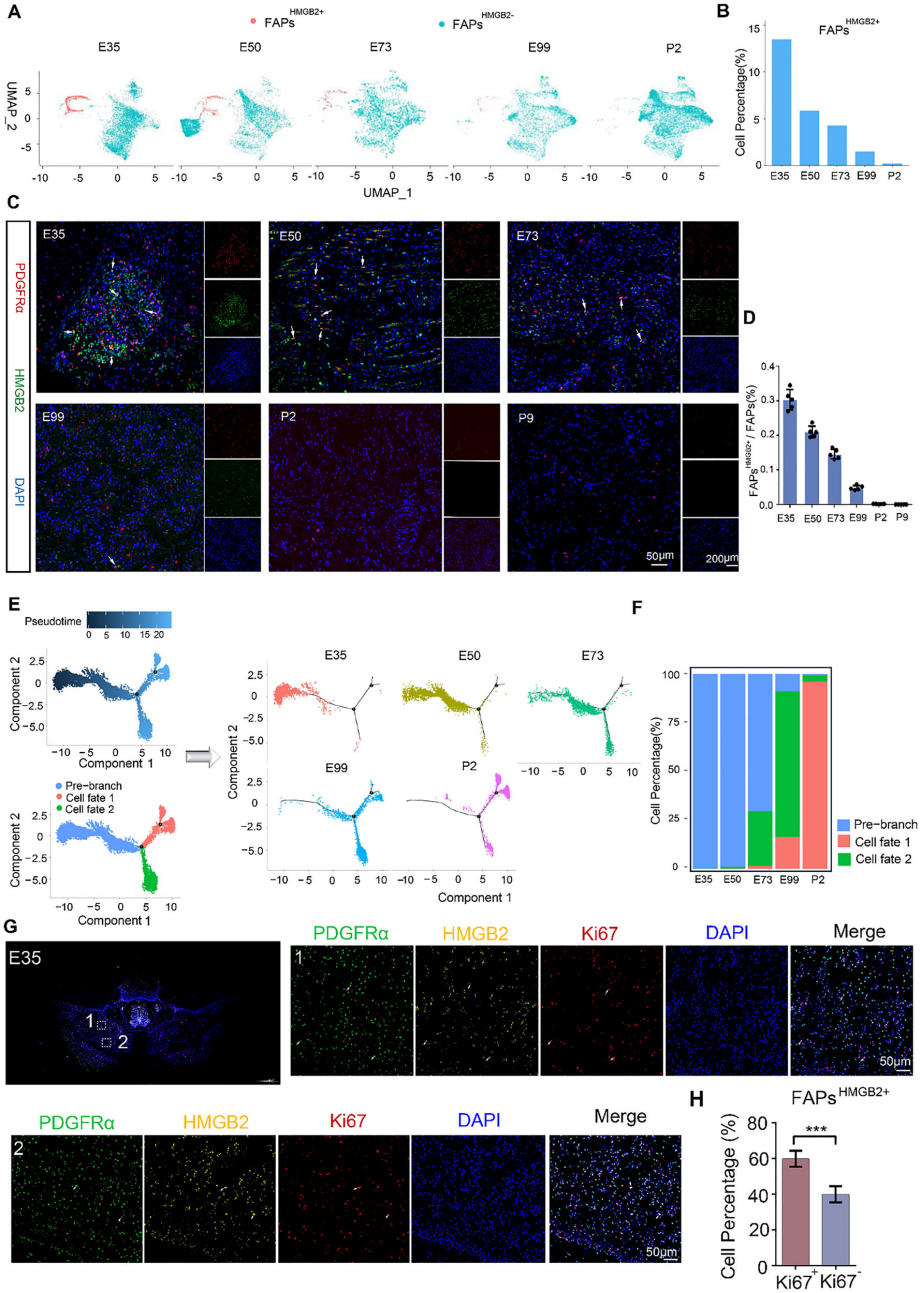
FAPs^HMGB2+^ is Predominantly Present During the Embryonic Stage of Porcine Skeletal Muscle Development. (A) UMAP plot showing the distribution of FAPs subpopulations in skeletal muscle at various developmental stages. (B) Proportion of FAPs^HMGB2+^ among total FAPs at each developmental stage. (C and D) Immunofluorescence co‐staining of HMGB2 and PDGFRα on transverse paraffin sections of dorsal muscles from GDSS at E35, E50, E73, E99, P2, and P9, with quantification of the proportion of FAPs^HMGB2+^ among FAPs. (E) Visualization of differentiation trajectories of FAPs across developmental stages. (F) Proportions of FAPs in three cell states (Pre‐branch, Cell fate 1, and Cell fate 2) in skeletal muscle at each developmental stage. (G) Multiplex immunofluorescence staining for PDGFRα, HMGB2, and Ki67 was performed on early‐stage skeletal muscle samples (E35). (H) The proportion of Ki67‐positive cells within the FAPsHMGB2^+^ population was quantified in early‐stage skeletal muscle samples (E35).

To further investigate the dynamic changes in FAP numbers, the relative proportions of FAP subgroups were analyzed across different developmental stages. The results demonstrated a decreasing trend in the proportion of FAPs^HMGB2+^ along with the progression of skeletal muscle development, with levels approaching zero at postnatal day 2 (P2) (Figure 3A,B). This finding was validated through immunofluorescence staining of the longissimus dorsi muscle samples collected throughout the entire intermuscular adipocyte development period (E35, E50, E73, E99, P2, and P9), demonstrating consistency with the ScRNA‐seq data (Figure 3C,D). Multiplex staining of Ki67, PDGFRα, and HMGB2 confirmed that FAPs^HMGB2+^ cells exhibit high proliferative activity during the embryonic stage in pigs.

Additionally, the distribution of FAPs in three cell states (Pre‐branch, Cell fate 1, and Cell fate 2) across different developmental stages showed that at embryonic day 35 (E35), 100% of FAPs were located in the Pre‐branch state. Along with the skeletal muscle development, the proportion of FAPs in the Pre‐branch state decreased, reaching zero at P2 (Figure 3E,F). Collectively, these results confirm that FAPs^HMGB2+^ predominantly exist during the embryonic period, indicating that the differentiation potential of FAPs is largely established during this period.

### 2.4 FAPs^HMGB2+^ Supplement the Loss of FAPs During Muscle Regeneration

**FIGURE 4:**
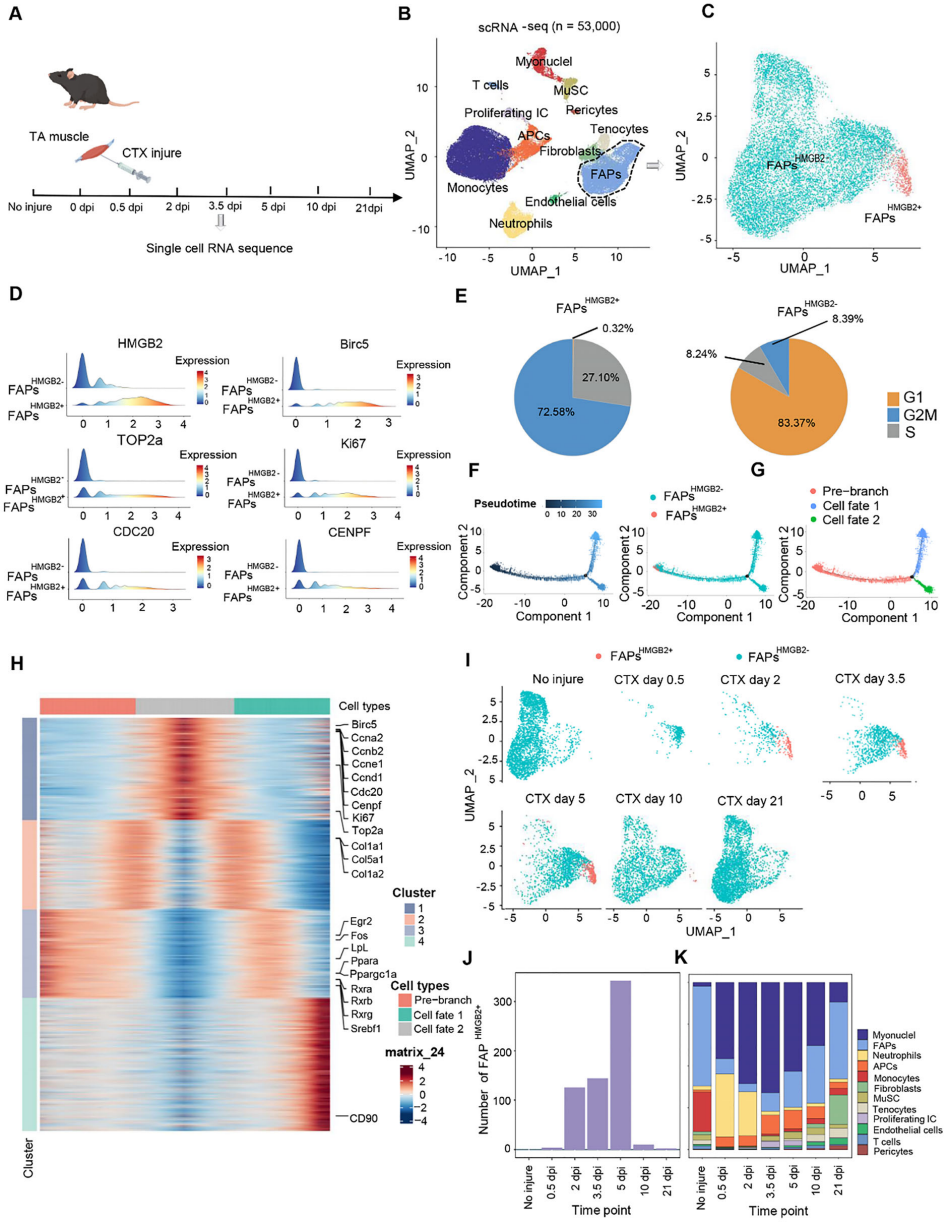
FAPs^HMGB2+^ compensates for the loss of FAPs during muscle Regeneration in Mice. (A) Schematic representation of muscle preparation, single‐cell isolation, and ScRNA‐seq procedures. (B) Graph‐based clustering of isolated single cells reveals distinct clusters corresponding to different cell populations. (C) Graph‐based clustering analysis identifies two subclusters within the FAPs population. (D) Expression of representative genes in the FAPs ^HMGB2+^. (E) Proportion of cell cycle phases in different FAPs subpopulations. (F and G) Pseudotime analysis of FAPs was performed using Monocle 2, identifying three different cell states (Pre‐branch, Cell fate 1, and Cell fate 2). The distributions of cell states are presented along pseudotime flows. Each dot represents a cell. (H) Heatmap showing the expression changes of the top 2,743 differentially expressed genes (DEGs) in pseudotemporal order, with DEGs cataloged into four major clusters characterized by distinct patterns. (I) UMAP plot showing subpopulation distribution of FAPs in skeletal muscle at various stages of muscle injury. (J) The quantity of FAPs^HMGB2+^ in skeletal muscle at various stages of muscle injury. (K) The proportion of each cell cluster in skeletal muscle at various stages of muscle injury.

During muscle injury, FAPs are typically activated [35]. To assess the involvement of FAPs^HMGB2+^ in this process, single‐cell RNA sequencing data from the GEO database (GSE138826) were analyzed, covering seven time points: uninjured, and 0.5, 2, 3.5, 5, 10, and 21 days post‐injury (Figure 4A). Following quality control, 53 000 cells were obtained. The data were normalized and visualized using UMAP, revealing 12 distinct cell clusters (Figure 4B; Figure S3A,B). A bubble plot was utilized to illustrate the marker genes with high expression in each cell cluster (Figure S3C).

The analysis focused on the FAPs cluster, revealing a subpopulation of FAPs marked by HMGB2, characterized by elevated levels of genes associated with cell proliferation (Figure 4C,D). The cell cycle analysis and GO enrichment results were similar to those observed in pig embryonic FAPs^HMGB2+^ (Figure 4E; Figure S3E), suggesting that FAPs^HMGB2+^ is also involved in the proliferation of FAPs during muscle regeneration. Pseudotime trajectory analysis using Monocle2 revealed that the developmental trajectory of FAPs during muscle regeneration closely resembled the trajectory observed during embryonic development (Figure 4F–H; Figure S3F). However, compared to embryonic cells, the Cell fate 2 branch of cells in muscle regeneration does not have the ability to produce collagen, but rather participates in promoting myoblast proliferation, as shown by GO enrichment analysis (Figure 4H; Figure S3F).

Cluster proportion analysis during muscle regeneration showed a significant decrease in FAPs from the uninjured state to day 2 post‐injury. Over time, the proportion of FAPs gradually recovered, returning to pre‐injury levels by day 21 (Figure 4K). These findings were further validated using the public dataset GSE143437 (Figure S5A–C). Correspondingly, FAPs^HMGB2+^ emerged on day 2 post‐injury, peaking on day 5 (Figure 4J). As the FAP pool in skeletal muscle was replenished, the number of FAPs^HMGB2+^ declined, eventually disappearing by day 21 post‐injury (Figure 4I). These findings suggest that muscle injury activates FAPs, resulting in the emergence of a distinct FAP subpopulation (FAPs^HMGB2+^) during muscle regeneration, thereby maintaining the homeostasis balance of the FAP pool in skeletal muscle.

### 2.5 HMGB2 Determines the Proliferative Potential of FAPs

**FIGURE 5:**
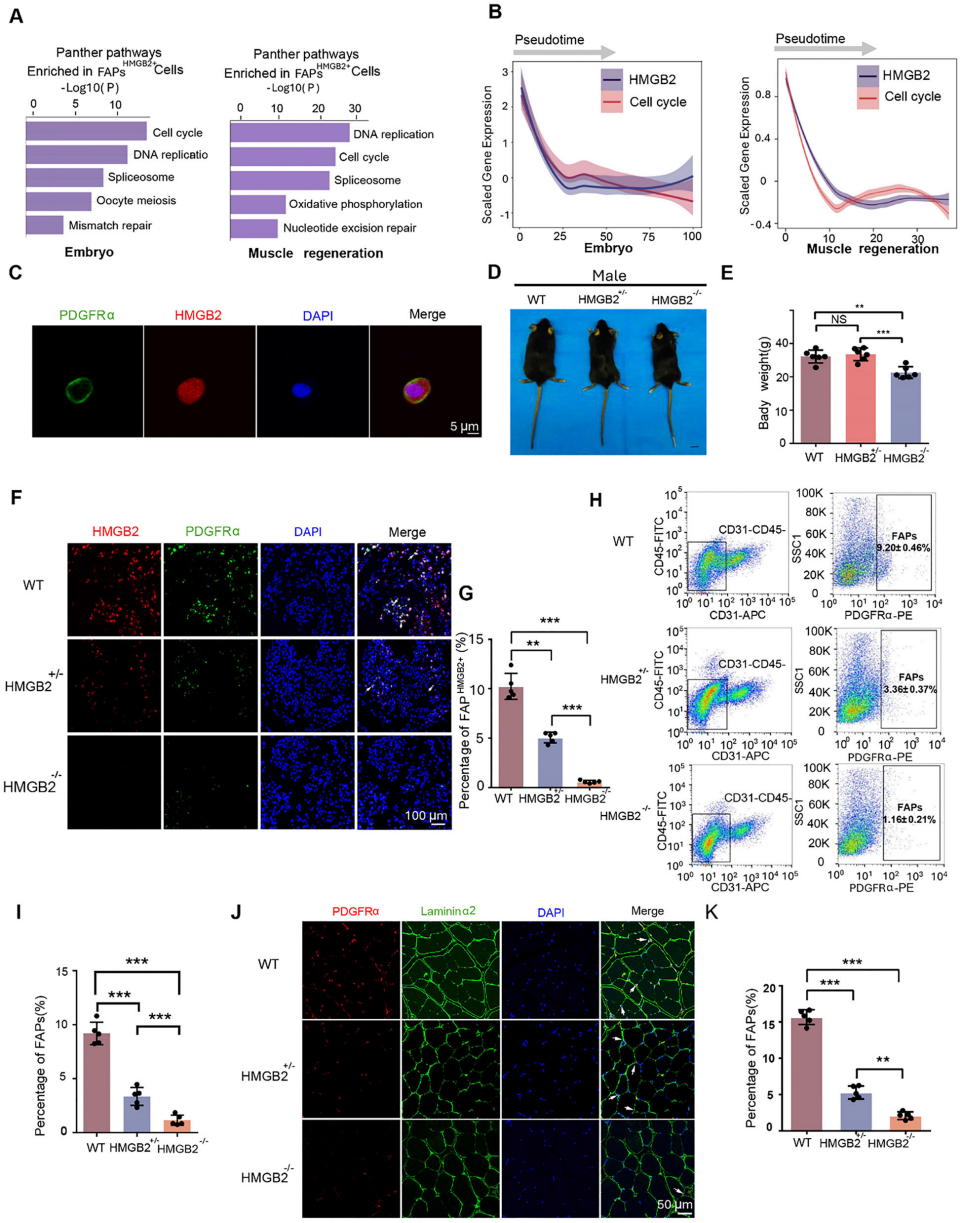
HMGB2 regulates the proliferative ability of FAPs. (A) KEGG pathway analysis of FAPs^HMGB2+^ during embryonic development and muscle injury. (B) Trends in the expression of HMGB2 and the Cell Cycle pathway. (C) Immunofluorescence co‐staining of PDGFRα and HMGB2 in mouse FAPs (CD31‐CD45‐PDGFRα+) cultured in vitro. (D) Representative images of 8‐week‐aged control HMGB2^+/−^and HMGB2^−/−^ male mice. Scale bar = 1 cm. (E) The body weight of male mice with different genotypes at 8 weeks of age (n = 6 for each genotype). (F) Immunofluorescence co‐staining of HMGB2 and PDGFRα in muscle at embryonic day 14 (E14). (G) Proportion of FAPs^HMGB2+^ among different genotypes. (H) Quantification of FAPs in tibialis anterior (TA) muscles of 8‐week‐old mice with different genotypes through flow cytometry. (I) Proportion of FAPs in TA muscles of 8‐week‐old mice with different genotypes. (J) Identification of FAPs content in TA muscles of 8‐week‐old mice with different genotypes based on immunofluorescence staining. (K) Quantification of FAPs content.

The function of the FAPs^HMGB2+^ subpopulation was investigated by conducting KEGG pathway analysis on genes expressed in these cells that exist in both embryonic development and muscle regeneration. The results indicated enrichment in pathways related to “Cell cycle” and “DNA replication” (Figure 5A). Further investigation revealed a strong correlation between HMGB2 expression pattern and the Cell cycle pathway during the developmental trajectory of FAPs (Figure 5B). Immunofluorescence staining confirmed HMGB2 localization in both the cytoplasm and nucleus of FAPs (Figure 5C).

To further investigate the role of HMGB2 in regulating the proliferative capacity of FAPs, HMGB2 knockout mice were generated (Figure S6). Western blot and qPCR results confirmed the successful knockout (Figure 5D; Figure S7B,C). Muscle samples from wild‐type (WT), HMGB2^+/−^, and HMGB2^−/−^ mice were collected at embryonic day 14 (E14). Immunofluorescence co‐staining for HMGB2 and PDGFRα revealed a significant reduction in FAPs^HMGB2+^ numbers both in HMGB2^+/−^ and HMGB2^−/−^ mice compared to WT controls, with FAP^HMGB2+^ being more abundant in HMGB2^+/−^ mice than in HMGB2^−/−^ mice. These findings highlight the role of HMGB2 in promoting FAPs proliferation (Figure 5F,G).

Flow cytometry was used to assess the FAPs content in the TA muscle of 8‐week‐old mice with different genotypes. A significant decrease in FAPs content was observed in HMGB2 knockout mice, with a gradient reduction across different genotypes, consistent with the trend observed in embryonic FAPs^HMGB2+^ (Figure 5H,I). This was further validated by immunofluorescence staining (Figure 5J,K).

The depletion of FAPs impaired skeletal muscle development. Notably, HMGB2^−/−^ mice, especially males, showed significant decreases in body weight, TA muscle size, and muscle strength compared to WT mice (Figure 5E; Figure S7D–M). Further, no significant differences were observed between HMGB2^+/−^ and WT mice in these parameters (Figure S7D–M), indicating that a moderate reduction in FAPs does not adversely affect skeletal muscle development.

### 2.6 HMGB2 Regulates the Deposition of Adipose and Collagen in Skeletal Muscle

Decline in proliferative capacity of FAPs due to HMGB2 knockout led to decreased FAPs content in the skeletal muscles of HMGB2^+/−^ and HMGB2^−/−^ mice. This observation raises a new question: whether the reduction of FAPs would influence the potential for fat infiltration and muscle fibrosis in skeletal muscle. To explore this, models of muscle injury, aging, and diabetes models were established using WT, HMGB2^+/−^, and HMGB2^−/−^ mice.

**FIGURE 6:**
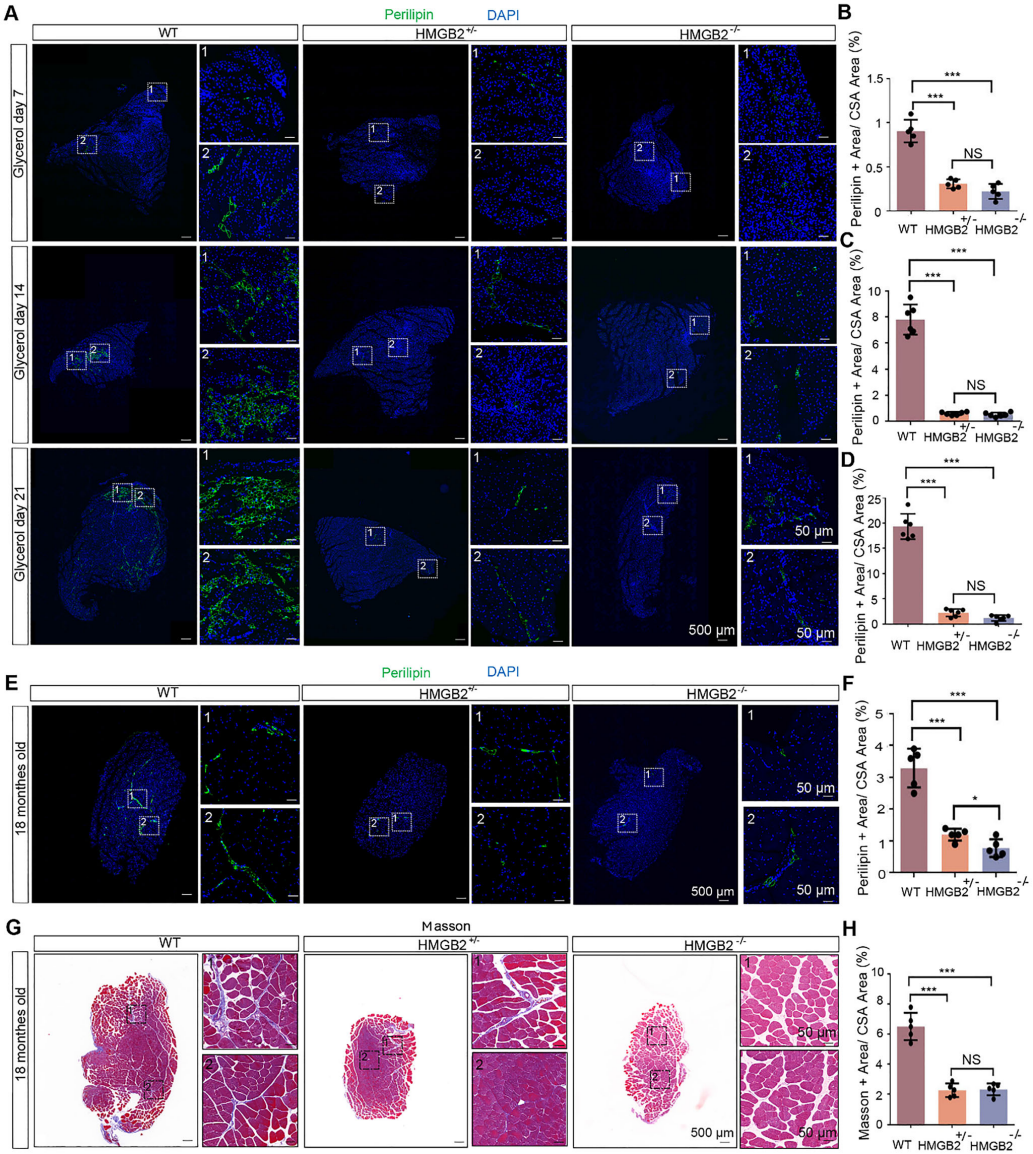
The Effect of Reduced FAPs in Skeletal Muscle on Muscle Injury and Aging. (A) Perilipin immunofluorescence staining was used to identify fat infiltration in skeletal muscle of WT, HMGB2^+/−^, and HMGB2^−/−^ mice at 7, 14, and 21 days post‐glycerol injury. (B) Quantification of Perilipin‐positive areas in muscle cross‐sections at 7 days post‐injury (*n* = 3). (C) Quantification of Perilipin‐positive areas in muscle cross‐sections at 14 days post‐injury (*n* = 3). (D) Quantification of Perilipin‐positive areas in muscle cross‐sections at 21 days post‐injury (*n* = 3). (E) Evaluation of fat infiltration in skeletal muscle of 16‐month‐old mice using Perilipin immunofluorescence staining. (F) Percentage of Perilipin‐positive areas in muscle cross‐sections in the aging model (*n* = 3). (G) Assessment of collagen deposition in muscle using Masson staining (aging model, 16 months old). (H) Percentage of Masson‐positive areas in muscle cross‐sections in the aging model (*n* = 3).

In the muscle injury model, fat content in the TA muscle was assessed at 7, 14, and 21 days post‐injury. Immunofluorescence staining for Perilipin showed a significant reduction in adipocyte numbers in the TA muscles of HMGB2^+/−^ and HMGB2^−/−^ mice compared to WT controls (Figure 6A–D). Minimal differences in adipocyte numbers were observed between HMGB2^+/−^ and HMGB2^−/−^ mice, indicating that even a modest reduction in FAPs decreases the capacity for fat infiltration in skeletal muscle. Similarly, adipocyte numbers and muscle fibrosis were assessed in aging mice of all three genotypes, with the observed changes in fat infiltration capacity aligning with those seen in the aging model (Figure 6E,F). A significant reduction in muscle fibrosis was also observed in the TA muscles of HMGB2^+/−^ and HMGB2^−/−^ mice (Figure 6G,H).

**FIGURE 7:**
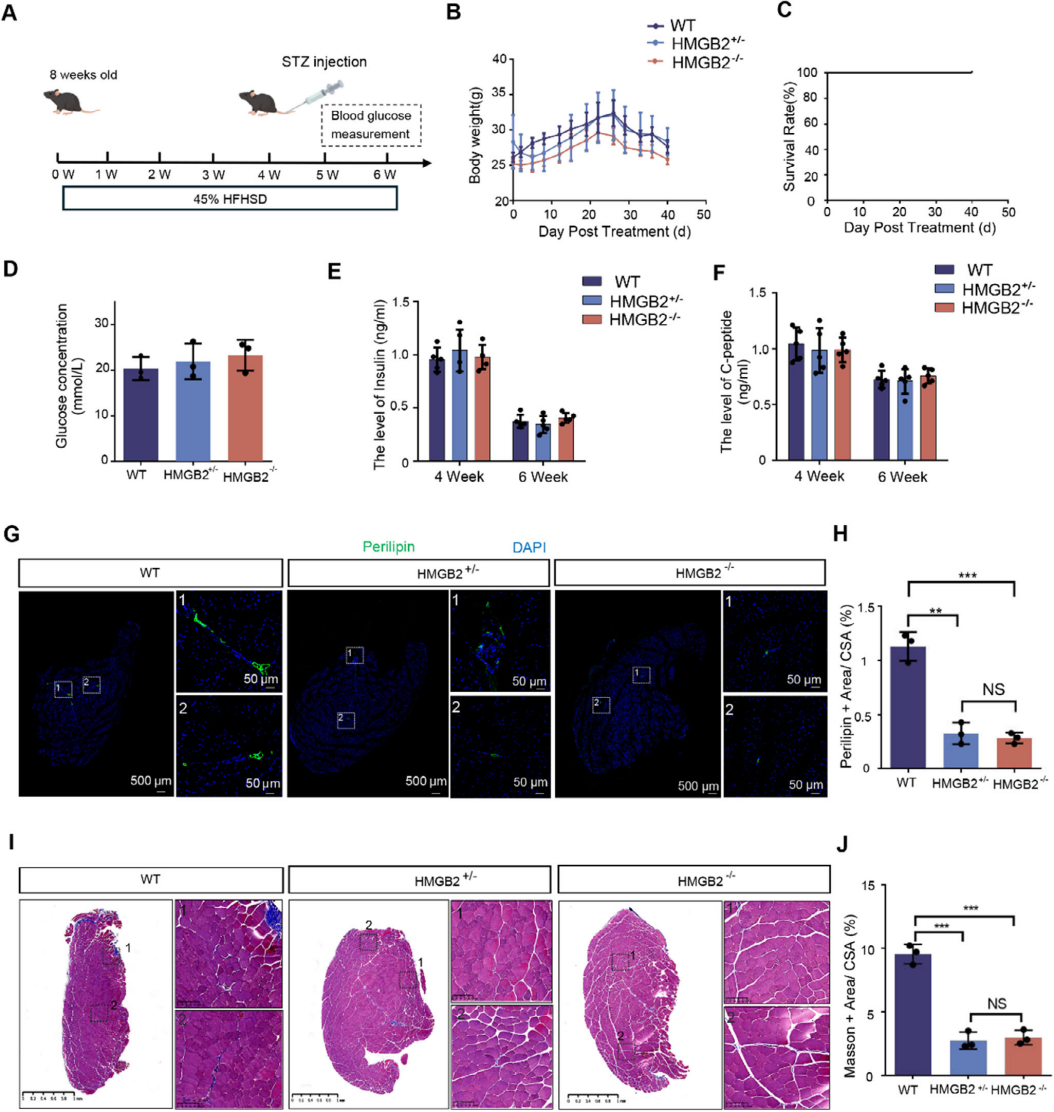
The Impact of Reduced FAPs in Skeletal Muscle on Diabetes‐model Mice. (A) Induction of diabetes‐model mellitus in mice using streptozotocin (STZ). (B) Body weight changes during the induction period. (C) Survival rate analysis during model induction. (D) Measurement of blood glucose concentration via tail vein sampling at 6 weeks post‐induction. (E) Fasting insulin levels in mice after 4 weeks of high‐fat diet and 2 weeks after STZ injection. (F) C‐peptide levels in mouse blood after 4 weeks of high‐fat diet and 2 weeks after STZ injection. (G) Evaluation of fat infiltration in skeletal muscle using Perilipin immunofluorescence staining. (H) Percentage of Perilipin‐positive areas in muscle cross‐sections (*n* = 3). (I) Assessment of collagen deposition in muscle using Masson staining. (J) Percentage of Masson‐positive areas in muscle cross‐sections (*n* = 3).

To assess whether these findings are applicable to muscle atrophy in a diabetes model, a diabetes model was induced using streptozotocin (STZ) (Figure 7A–F). Six weeks post‐induction, blood glucose levels confirmed successful model establishment (Figure 7D‐F). No abnormalities were observed in any of the mice during the modeling process (Figure 7B,C). Subsequent assessments of fat infiltration and muscle fibrosis in skeletal muscles revealed significant reductions in the TA muscle of HMGB2^+/−^ and HMGB2^−/−^ mice, consistent with the trends observed in other models (Figure 7 G‐J). These findings suggest that reducing FAPs numbers effectively mitigates fat infiltration and muscle fibrosis.

### 2.7 HMGB2 Promotes FAPs Proliferation by Targeting RAD21

**FIGURE 8:**
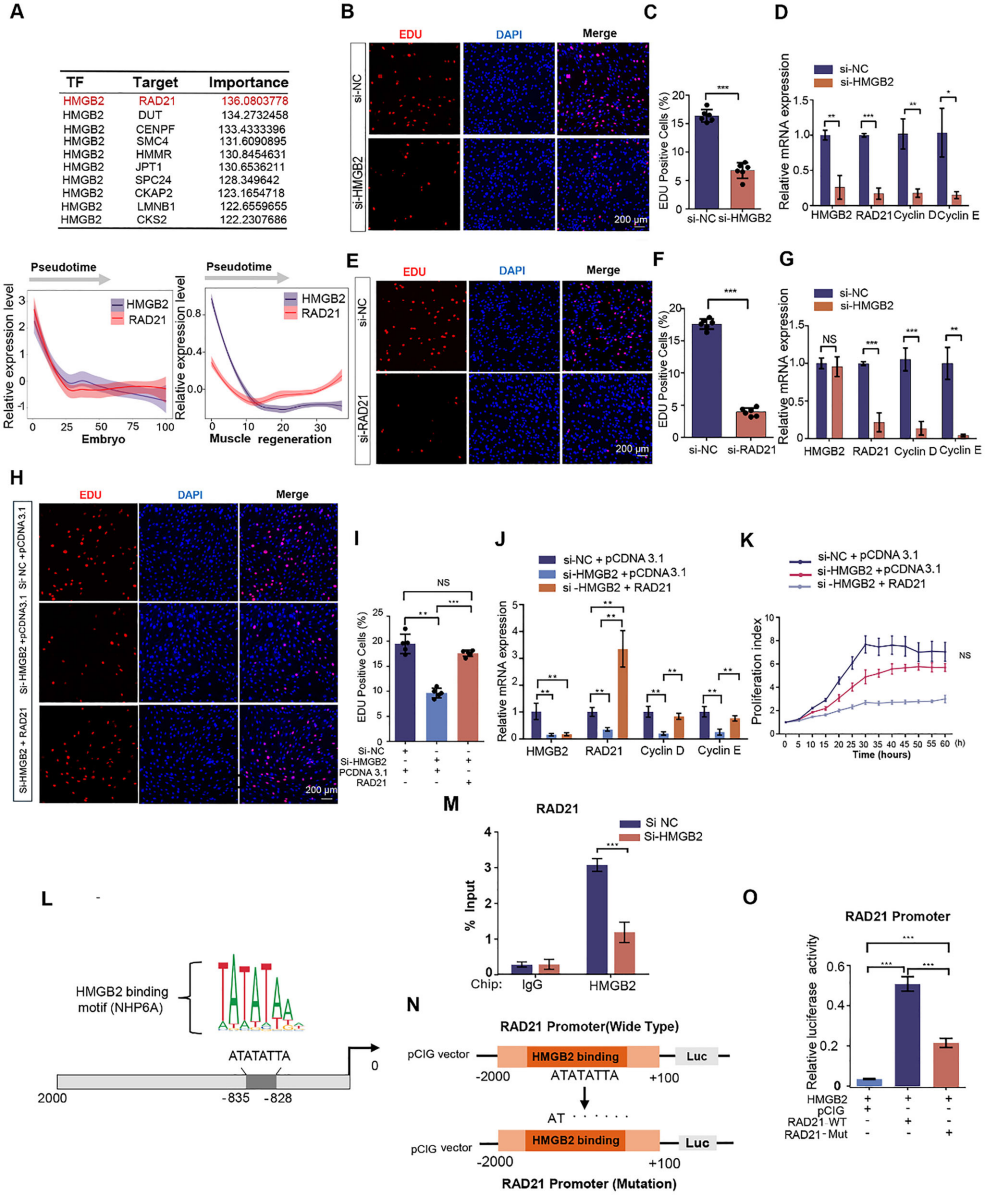
HMGB2 directly binds to the RAD21 promoter and activates its transcription. (A) Trends in HMGB2 and RAD21 expression in FAPs developmental trajectories. (B, C) EDU staining of mouse FAPs after transfection with si‐HMGB2. (D) The mRNA levels of HMGB2, RAD21, Cyclin E1, and Cyclin D1 were detected by qPCR (*n* = 3) after transfection with si‐HMGB2. (E, F) EDU staining of mouse FAPs after transfection with si‐RAD21. (G) The mRNA levels of HMGB2, RAD21, Cyclin E1, and Cyclin D1 were detected by qPCR (*n* = 3) after transfection with si‐RAD21. (H) EDU staining of mouse FAPs after transfection with si‐HMGB and pCDNA3.1‐RAD21. (I) Quantification of EDU^+^ cells. (J) The mRNA levels of HMGB2, RAD21, Cyclin E1, and Cyclin D1 were detected by qPCR (*n* = 3). (K) Real‐time proliferation index of FAPs. (L) Prediction of HMGB2 binding sites within the RAD21 promoter region (−2000 to +0bp) using the JASPAR database. (M) ChIP‐qPCR analyses of HMGB2 enrichment on RAD21 promoter in control and KLF4 knockdown cells. Data were normalized as a percentage of the input. (N) Experimental design involving amplification of the RAD21 promoter sequence from −2000 to +0 bp, deletion of the predicted binding region "ATATTA," and insertion into a promoter‐driven dual luciferase reporter plasmid pCIG vector carrying both firefly luciferase (Luc) and sea pansy luciferase (RLuc) (O) Dual luciferase reporter gene assay performed in 293T cells transfected with reporter plasmids containing wild‐type or mutant RAD21 promoters, along with pCDNA3.1‐HMGB2 vector (*n* = 3).

Considering that the above results have demonstrated HMGB2 as a key regulator of fat infiltration, we employed SCENIC to predict its target genes and identified RAD21, a gene crucial for DNA replication, as a potential downstream target regulated by HMGB2. Moreover, the expression patterns of HMGB2 and RAD21 exhibit a strong correlation (Figure 8A). To further verify their regulatory relationship, RAD21 was knocked down in FAPs, which led to a significant reduction in FAP proliferation, confirming its critical role in this process (Figure 8E–G). In contrast, overexpression of RAD21 in HMGB2‐knockdown FAPs restored proliferative capacity, as shown by EDU staining and real‐time proliferation assays (Figure 8H–F). At the mRNA level, the expression of proliferation‐related genes Cyclin D and Cyclin E was also rescued (Figure 8D,G,J). These results demonstrate that RAD21 functions downstream of HMGB2 and mediates its regulatory effect on FAP proliferation and expansion.

Potential HMGB2 binding sites within the RAD21 promoter region, spanning from ‐835 bp to ‐828 bp, were predicted using the JASPAR database (Figure 8L). ChIP assays further confirmed that HMGB2 binds directly to the RAD21 promoter (Figure 8M). To validate this binding site's role in RAD21 transcriptional regulation, a promoter sequence containing the HMGB2 binding motif was cloned into a dual‐luciferase reporter vector pCIG208 [36] (Figure 8N). Mutation of the binding motif significantly decreased RAD21 transcriptional activity, demonstrating that HMGB2 directly targets RAD21 (Figure 8O).

## 3 Discussion

Fat infiltration in skeletal muscle is strongly linked to various aspects of human health and meat quality in animal husbandry [37]. However, the underlying mechanisms of adipocyte development within skeletal muscle remain unclear. This study identifies the presence of intermuscular adipocytes after birth (about 36 h postnatally) and, using ScRNA‐seq, maps their developmental trajectory. A distinct cell type, FAPs^HMGB2+^, was identified, primarily present during the embryonic period. These cells play a crucial role in regulating FAPs abundance and are activated during muscle injury to replenish the loss of FAPs. HMGB2 was identified as a key regulatory gene that influences the proliferative capacity of FAPs. Proper regulation of FAPs abundance in skeletal muscle may reduce fat infiltration and muscle fibrosis while promoting the normal development and function of skeletal muscle.

The timing of adipocyte commitment and differentiation varies among different fat depots [38, 39]. In mice, subcutaneous adipocytes begin to differentiate at embryonic day 14 [40], visceral adipocytes at embryonic days 16–18 [41], while the differentiation timeline of intermuscular adipocytes remains previously unknown. Skeletal muscle development is divided into two stages: the formation of primary (embryonic days 35 and 50) and secondary muscle fibers (embryonic days 73 and 99) [42]. Postnatal days 2 and 9 correspond to the neonatal period, day 80 to the juvenile period, and day 180 to adulthood [43]. This study examined eight developmental stages and, through histological staining and bulk RNA sequencing, identified the early postnatal period as a starting window for the differentiation of intermuscular adipocytes in skeletal muscle.

As non‐myogenic mesenchymal stem cells, FAPs lack myogenic potential but proliferate rapidly in response to muscle injury, promoting myogenesis in vitro [5]. FAPs possess the capacity and also exhibit potential for both fibrosis and adipogenesis, suggesting cellular heterogeneity [4]. This study investigated the developmental trajectories of FAPs during both embryogenesis and muscle regeneration, revealing dual potentials for adipogenesis and collagen synthesis. A specific subpopulation of proliferative cells, FAPs^HMGB2+,^ was identified in both contexts.

Under normal or non‐injury conditions, mesenchymal progenitor cells (MSCs) in skeletal muscle generally remain in a quiescent state [44]. This study identified that FAPs^HMGB2+^ are predominantly present during embryonic development and become activated in response to muscle injury. In steady‐state muscle, FAPs also remained quiescent, consistent with previous research [44]. During embryogenesis, FAPs^HMGB2+^ played a crucial role in establishing the size of the FAPs pool. In response to muscle injury, these cells were activated to replenish the FAPs, thereby preserving the homeostasis of the FAPs pool. This suggests that the proliferative capacity of embryonic FAPs^HMGB2+^ is a critical determinant of the FAPs pool size, with potential implications for modulating this capacity to influence the FAPs pool. This hypothesis is further supported by findings from HMGB2 knockout mice utilized in this study.

The relationship between fat infiltration in skeletal muscle and the size of the FAPs pool is well established [22, 23, 44, 45, 46]. Early‐stage type 2 diabetes, for example, is associated with a significant increase in the number of FAPs within skeletal muscle [47]. Based on this correlation, it was hypothesized that the size of the FAPs pool impacts the potential for fat infiltration and fibrosis in skeletal muscle. Supporting this concept, UCP1 knockout‐induced FAP reduction models [48] and D2 knockout‐induced FAP reduction models [49] consistently demonstrate that decreased FAP abundance markedly suppresses fatty infiltration and collagen deposition in injured or diseased muscle. To further confirm this conclusion, fat infiltration and fibrosis were examined in HMGB2 knockout mice subjected to muscle injury, type 2 diabetes, and aging. As expected, a reduction in the FAPs pool size diminished the phenotypes of fat infiltration and fibrosis. FAPs in skeletal muscle are not only linked to fat infiltration and fibrosis but also promote myocyte proliferation, influencing muscle development [4, 37, 50]. In this study, HMGB2^−/−^ mice exhibited impaired muscle fiber development and decreased limb strength. Notably, HMGB2^+/−^ mice showed no adverse effects on muscle development, suggesting that a moderate reduction in the FAP pool does not impede skeletal muscle development.

HMGB2, a non‐histone protein belonging to the High‐Mobility Group (HMG) superfamily, is part of the HMGB domain proteins [29, 30]. HMGB2 is primarily involved in DNA binding activity and chromatin structure organization. Subsequent research has revealed its participation in various biological processes, including chromatin remodeling, transcription, and DNA repair [31]. HMGB2 has also been shown to regulate ovarian follicle development and the senescence‐associated secretory phenotype (SASP) [51, 52]. Furthermore, HMGB2 has been shown to regulate the potential for fat infiltration during muscle injury [30]. In this study, HMGB2 was found to be specifically highly expressed in the FAPs^HMGB2+^ cluster and was shown to target the RAD21 promoter region, thereby regulating the proliferation of FAPs. Analysis of the Tabula Muris database confirmed that HMGB2 is broadly expressed across multiple cell types (Figure S6), indicating that global deletion may affect various lineages and developmental processes beyond FAPs. Although HMGB2 knockout mice exhibited reduced body weight and subcutaneous fat (Figure S7), they remained healthy, viable, and fertile, indicating that HMGB2 is not essential for mouse survival or that its function may be compensated through alternative mechanisms. These healthy and viable mice therefore provide a valuable resource for investigating the function of cell‐type‐specific HMGB2 deletion. In this study, we focused on the role of FAPs in intermuscular adipogenesis. Both in vivo and in vitro experiments consistently demonstrated that HMGB2 deletion led to a significant reduction in FAP numbers. It is worth noting that HMGB2 may also influence the physiological functions of other cell types, including myogenic cells, as reported in our previous study [53].

In summary, this study is the first to identify the early postnatal period as a critical stage for the initial differentiation of intermuscular adipocytes. The first developmental atlas of embryonic FAPs in porcine skeletal muscle was constructed, revealing a novel cell type characterized by high HMGB2 expression (FAPs^HMGB2+^). These findings demonstrate that HMGB2 expression levels regulate the size of the skeletal muscle FAPs pool through the HMGB2/RAD21 axis, thereby determining the adipogenic potential of intermuscular fat postnatally. This discovery not only advances our understanding of skeletal muscle adipogenesis but also highlights the HMGB2/RAD21 axis as a promising regulatory pathway for modulating intramuscular fat deposition in livestock breeding. Future research could further investigate how these insights can be applied to optimize therapeutic interventions and breeding practices, addressing the need for healthy skeletal muscle and high‐quality meat, respectively.

## 4 Materials and Methods

### 4.1 Ethics Statement

All procedures involving animals were performed in accordance with the guidelines established by the China Council on Animal Care. The study protocol received approval from the Sun Yat‐Sen University Animal Care and Use Committee (Approval ID: SYSU‐IACUC‐2020‐B0614).

### 4.2 Animals

Male Guangdong small‐eared spotted pigs (GDSS), matched for age and physical condition, were utilized at eight developmental stages: four prenatal stages (E35, E50, E73, E99) and four postnatal stages (P2 (36 h after birth), P9, P80, P180). Prior to euthanasia, pigs were fasted for 12 h. The gender of pig fetuses at E35 was determined using a PCR‐based method [54], as sexual characteristics were not visible before day 49 post‐conception.

Validation experiments were performed using hmgb2^−/−^ mice generated via the CRISPR/Cas9 system. Cas9/sgRNAs targeting exons 2–5 of hmgb2 were injected into zygotes (Table S3), which were transplanted into the oviducts of female C57BL/6J mice. Positive F0 mice were crossed with wild‐type C57BL/6J mice to produce F1 mice with stable genotypes (Table S4). Homozygous hmgb2^−/−^ mice were generated from these F1 mice [55]. Mice were housed under specific‐pathogen‐free (SPF) conditions, maintained on a 12‐h light/dark cycle, with unrestricted access to food and water.

### 4.3 RNA‐Seq Library Construction and Sequencing

Total RNA was extracted using Trizol reagent (Invitrogen, Carlsbad, CA, USA) following the manufacturer's instructions. RNA from skeletal muscle at different developmental stages was used for library construction. Genomic DNA was removed using the DNase I enzyme. RNA quality and concentration were assessed using agarose gel electrophoresis and a 2100 Bioanalyzer (Agilent Technologies), ensuring RNA integrity numbers ≥7.0 and 28S:18S ratios ≥0.7. RNA‐seq libraries were constructed according to the Illumina protocol.

Polyadenylated RNA was isolated using Oligo dT magnetic beads (Invitrogen, Carlsbad, CA, USA), reverse‐transcribed to synthesize first‐strand cDNA. Second‐strand cDNA was synthesized, followed by end‐repair, dA‐tailing, and adapter ligation. Fragments of suitable size were selected via agarose gel electrophoresis, enriched by PCR amplification, and assessed using an Agilent 2100 Analyzer. The libraries were then subjected to paired‐end sequencing with 90‐bp reads using an Illumina HiSeq 2000 sequencing system.

### 4.4 Transcriptome Data Analysis

Reference genome sequences and gene annotation files were obtained from Ensemble v95. RNA‐seq reads were aligned to the Sus scrofa reference genome (v11.1) [56] using TopHat v2.1 [57], allowing up to two mismatches (Tables S4 and S5). Gene expression levels were estimated by calculating RPKMs using HTSeq (version 0.6.1) [58]. K‐means clustering [59] was applied to identify expression patterns of differential genes across various developmental stages. Gene ontology (GO) enrichment analysis was performed to annotate the differentially expressed genes, with significant enrichment results refined based on an adjusted *P*‐value < 0.05.

### 4.5 Preparation of Single‐Cell Suspensions

Muscle tissues from three individuals from the same sow at stages E35, E50, E73, E99, and P2 were isolated and transferred to RPMI 1640 medium (GIBCO, 11875093) containing 10% fetal bovine serum (GIBCO, 1099‐141) on ice. Tissues were washed with PBS and subjected to enzymatic dissociation with 0.17% protease (Sigma–Aldrich, USA) and 0.28% collagenase‐type I solution (Sigma–Aldrich, USA). Cells were filtered through a 40‐µm cell strainer. Due to the lack of commercially available antibodies for flow sorting in porcine skeletal muscle, a previously described approach [60] was used to purify FAPs. The viability of single cells was assessed using trypan blue staining (Thermo Fisher Scientific, USA).

### 4.6 10× Genomics ScRNA‐Seq

Droplet‐based ScRNA‐seq datasets were generated using a Chromium system (10× Genomics, PN120263) following the manufacturer's instructions. After droplet generation, samples were transferred into pre‐chilled 8‐well tube strips (Eppendorf) for reverse transcription using a Veriti 96‐well thermal cycler (Thermo Fisher). cDNA was recovered using the Recovery Agent provided by 10× Genomics, purified with Silane Dynabeads (Thermo Fisher), and then amplified for 12 cycles. Cleanup was performed with SPRIselect beads (Beckman). The samples were diluted 1:4 and assessed for cDNA concentration using a Bioanalyzer (Agilent Technologies). Finally, cDNA libraries were prepared according to the Single Cell 3′ Reagent Kit v3 user guide, with adjustments to the PCR cycles based on cDNA concentration as recommended by 10× Genomics.

### 4.7 Single‐Cell RNA Sequencing Data Analysis

#### 4.7.1 Quality Control and Doublet Removal

The Digital Gene Expression (DGE) matrix was generated using the CellRanger (10× Genomics) analysis pipeline. The raw DGE matrix, which contains UMI counts per cell per gene, was processed using R software version 3.5.2 (https://www.r‐project.org/). To ensure high data quality, cells with fewer than 200 genes, cells with over 30 000 UMIs, and cells expressing 10% or more mitochondrial genes were excluded. Doublets identified by the Doublet Finder algorithm [61] and cells with more than 40 000 unique transcripts were also removed. Genes expressed in three or fewer cells were also excluded.

#### 4.7.2 Cell Clustering and Cell‐Type Annotation

Data normalization was performed using the Seurat R package (v3.1.1) with default parameters [62]. Cross‐Characterization Anchors (CCAs) were employed to integrate multiple samples. Dimensionality reduction was conducted using the top 30 principal components, followed by cell grouping through the Louvain algorithm (resolution = 0.6), resulting in 19 distinct gene clusters. Differential gene expression analysis was carried out using the Wilcoxon rank‐sum test, with a fold change threshold of 0.25 (log2) and a significance level of adjusted *p* < 0.05 for each group. Sub‐clustering analysis was conducted to investigate the heterogeneity within FAPs cluster.

#### 4.7.3 Pseudotime Trajectory Analysis

Pseudotime trajectory analysis was performed using Monocle v2.8.0 [63]. Cell differentiation datasets were generated based on cell IDs identified through the Seurat package. Dimensional reduction was performed using the DDRTree method to visualize the dataset. Cells were ordered based on global gene expression levels to delineate the trajectory of the dataset. Heatmaps were generated to depict smooth gene expression curves across pseudotime, illustrating gene expression trends.

### 4.8 Histological and Immunohistochemical Analysis

#### 4.8.1 Hematoxylin–Eosin (H&E) Staining

Fixed muscle tissues were embedded in paraffin, sectioned into 6 µm slices, and subjected to H&E staining. Sections were deparaffinized, rehydrated, and stained with hematoxylin for 15 min. Following a rinse with running tap water, sections were stained with eosin for 3–5 min, dehydrated, mounted, and imaged.

#### 4.8.2 Oil Red O Staining

Muscle cross‐sections were fixed with 4% paraformaldehyde for 30 min at room temperature and then stained with 60% saturated Oil Red O reagent (Sigma–Aldrich, USA) for 30 min. After staining, samples were washed three times with 40% ethanol and PBS for 2 min each and visualized using a Nikon brightfield microscope (Nikon, Japan).

#### 4.8.3 Polychromatic Immunofluorescence Staining

Polychromatic immunofluorescence staining was performed using a four‐color multiple fluorescent immunohistochemical staining kit (Absin, China, abs50012) and the Tyramide Signal Amplification (TSA) technique, following the manufacturer's instructions. Cultured cells and muscle cross‐sections were fixed in 4% paraformaldehyde for 20 min, permeabilized with 0.5% Triton X‐100/PBS for 15 min, and blocked with 5% BSA/PBS for 2 h. Samples were then incubated with primary antibodies for 1 h at room temperature, followed by washing in PBST. Horseradish peroxidase‐labeled secondary antibodies were applied, followed by treatment with a fluorescent dye diluted in signal amplification reagent. For multiple fluorescent staining, antigen retrieval was performed between each staining cycle to remove bound antibodies. Finally, nuclei were stained with DAPI, and images were acquired using a fluorescent microscope (Nikon, Japan). Antibodies used are listed in Table S1.

#### 4.8.4 Masson Staining

Mouse TA muscle tissue was fixed, embedded in paraffin, sectioned into 6 µm slices, and stained with Masson trichrome (Baso, BA‐40798) following dewaxing. Observations were made using an inverted microscope (Nikon, Japan). The percentage of collagen fibrosis area was analyzed across all visual fields in each slice using Image‐Pro Plus 6 software (Media Cybernetics, Bethesda, USA).

### 4.9 FAPs Isolation by FACS from Mouse TA Muscle

The tibialis anterior (TA) muscle was minced in 200 µL of digestion buffer (DMEM/F12 and 2.8 mg/mL collagenase I) within a 1.5 mL tube, then transferred to a 50 mL Falcon tube containing 10 mL of digestion buffer. The tissue was incubated in a 37°C shaking water bath for 1 h. Following digestion, the solution was filtered through a 100 µm cell strainer, diluted to 30 mL with 2% FBS in PBS, and centrifuged at 600 × g for 5 min. The supernatant was discarded, and red blood cells were lysed using 1 mL RBC Lysis Buffer (Sigma, USA) from the stromal vascular fraction (SVF) pellet. The mixture was diluted to 10 mL with 2% FBS/PBS, filtered through a 40 µm cell strainer, and centrifuged at 600 × g for 5 min. After removing the supernatant, the cells were resuspended in a blocking buffer (2% FBS/PBS with anti‐mouse CD16/CD32 Fc Block at a 1:200 dilution). Primary antibodies were then added to the cell suspension and incubated for 30 min at 4°C in the dark (see Table S1). After washing with 2% FBS/PBS, cells were resuspended for sorting and collection using a Beckman CytoFlex instrument. Flow cytometry plots were generated using FlowJo (V10).

### 4.10 Cell Culture and Proliferation

Sorted FAPs (CD31‐CD45‐PDGFRα+) were cultured in DMEM/F12 medium (Thermo Scientific, USA) supplemented with 20% fetal bovine serum (FBS; Gibco‐BRL, USA), 2 mm L‐glutamine (Gibco‐BRL, USA), and 5 ng/mL basic fibroblast growth factor (bFGF; PEPTECH, USA). Cultures were maintained in a 37°C incubator with 5% CO_2_. Cell proliferation was assessed using EdU staining and real‐time monitoring assays. EdU staining was conducted with the CellLight EdU DNA Cell Proliferation Kit (RiboBio, China) following standard protocols [64]. Real‐time cell proliferation was monitored using the xCELLigence RTCA system (ACEA Biosciences, CA, USA), with an initial seeding density of 5,000 cells. Proliferation was continuously monitored in E‐Plate 16 and analyzed using RTCA software 2.0.

### 4.11 RNA Interference and Overexpression

RNA interference and overexpression experiments were conducted to modulate gene expression in FAPs. Negative control siRNAs (siNC) and HMGB2 siRNAs (siHMGB2) were obtained from GenePharma Co., Ltd. (Shanghai, China) (see Table S2 for sequences). For overexpression, the coding sequences (CDSs) of the mouse RAD21 gene were inserted into the pcDNA3.1 vector (Invitrogen) to create the RAD21 expression vector (pcDNA3.1‐HMGB2). FAPs were seeded in 24‐well plates 12 h prior to treatment. Subsequently, transfection was performed using Lipofectamine 3000 (Invitrogen), introducing either siRNAs or plasmids into the cells.

### 4.12 Chromatin Immunoprecipitation (ChIP)

FAPs transfected with pcDNA3.1 vector or pcDNA3.1‐HMGB2 were cross‐linked with 1% formaldehyde for 8 min to create protein‐DNA complexes. Cell lysates were sonicated by Bioruptor (Covaris, USA) for 8 min to produce chromatin fragments of 200–300 bp. The clarified nuclear extracts were incubated with HMGB2 antibody overnight at 4°C, with IgG serving as a negative control. JASPAR 2018 (https://jaspar2018.genereg.net/matrix/MA0345.1/) was used to predict HMGB2 binding sites in the RAD21 promoter sequence. Specific qPCR primers for each predicted motif were designed using the NCBI database, and their specificity was verified using mouse genomic DNA. Precipitated chromatin DNA was analyzed via qPCR, and primers that successfully amplified DNA are listed in Table S2.

### 4.13 Vector Construction and Dual‐Luciferase Reporter Assay

The promoter sequences of the mouse RAD21 gene, including predicted HMGB2 binding motifs, were obtained from the NCBI database and amplified by PCR. The HMGB2 binding motif identified by ChIP‐qPCR was mutated, and the wild‐type and mutated promoters were inserted into the pCIG vector [36] between the Ampicillin and Luciferase restriction sites using the In‐Fusion HD Cloning kit (Takara Bio). When the density reached 70%, FAPs were transfected with 500 ng of the wild‐type or mutated promoter construct mixed with 50 ng of pcDNA3.1‐HMGB2 using Lipofectamine 3000 (Invitrogen). The medium was replaced after 12 h. Chemiluminescence was detected using the Dual Luciferase Reporter Assay System (Promega, Madison, WI), and promoter activity was determined by dividing the relative fluorescence value for firefly luciferase by that of Renilla luciferase.

### 4.14 RNA Extraction and Real‐Time Quantitative PCR

Total RNA was extracted from cultured cells using Trizol Reagent (Invitrogen), and cDNA synthesis was performed using StarScript II First‐strand cDNA Synthesis Mix (Genestar, Beijing, China) from 1 µg of total RNA. Real‐time quantitative PCR (qPCR) analyses were conducted on a LightCycler 480 II system (Roche, Basel, Switzerland) utilizing SYBR Green qPCR Mix (GDSBio, Guangzhou, China), with β‐actin serving as an internal control. Primers are listed in Table S2.

### 4.15 Skeletal Muscle Injury

Mice were anesthetized with isoflurane, and 50 µL of a 50% glycerol solution (Sigma–Aldrich, USA) was injected into the central two‐thirds of the TA muscle using an insulin syringe. The TA muscle was then manually pierced approximately 10 times with the same needle to promote dispersion of the injected glycerol.

### 4.16 Hang Test

Muscle strength in mice of different genotypes was assessed using a hang test. Mice were placed on a grid and allowed to hang in a suspended position. The duration of endurance, or time maintained in the suspended position, was recorded. This process was repeated six times to ensure the accuracy of the data.

### 4.17 Diabetes‐Model Mouse Model

Male mice, approximately 8 weeks old, were selected, with six mice chosen for each genotype. After a 3–5 day acclimatization period, a high‐fat, high‐sugar diet (45% HFHSD) was initiated on day 0. When the mice reached a weight of 30–35 g, low‐dose streptozotocin (STZ, 40mg/kg) was administered in small doses over five consecutive days. Postprandial blood glucose levels were measured during the first week after treatment. In addition, fasting serum insulin (PI602, Beyotime Biotech. Inc., Shanghai, China) and C‐peptide (EM0947, FinrTest, WuHan) levels were measured using ELISA kits to assess pancreatic β‐cell function. Mice were considered to have developed type 2 diabetes when fasting blood glucose exceeded 11.1 mmol/L and random blood glucose exceeded 16.7 mmol/L [65].

### 4.18 Western Blot

Proteins were extracted from fresh muscle tissues in RIPA buffer containing 1 mM PMSF (Genstar, China) by SDS polyacrylamide gel electrophoresis. The proteins from the gel were then transferred onto PVDF membranes (Millipore, USA). Subsequently, the membranes were blocked with 5% BSA in TBST (Tris‐buffered saline containing 0.1% Tween‐20) for 1 h at room temperature and then incubated with primary antibodies overnight at 4°C. The membrane was washed three times with TBST and incubated for 1 h with a secondary antibody conjugated to HRP for detection. Readouts were generated using enhanced chemiluminescence (FDbio, China). Antibodies were listed in Table S1.

### 4.19 Statistical Analysis

All experiments were conducted with a minimum of three biological replicates. Statistical analyses between different groups were performed using Student's t‐test or one‐way or two‐way ANOVA. Data are presented as mean ± SD, with statistical significance indicated as follows: ^*^ *P* < 0.05, ^**^ *P* < 0.01, ^***^ *P* < 0.001, and *P* ≥ 0.05: not significant (n.s.).

## Author Contributions

D.M. and X.T. designed and conceived the project. X.T. analyzed the ScRNA‐seq data. X.T., Z.L., T.D., and Q.Z. helped in sample collection and cell culture. Z.L., T.D., L.P., and R.X. performed RNA extraction, qPCR, and immunofluorescence assays. J.L., X.C., Y.L., Y.L., X.L., L.C., and Q.F. contributed to data processing, discussions, and advice. Y.C., X.L., and D.M. gave financial support. X.T. and D.M. wrote and revised the manuscript. Y.C. and X.L. were responsible for the correction. All authors read and reviewed the final manuscript.

## Funding

Agricultural Science and Technology Maior Project, National Natural Science Foundation of China (32030102, 323B2058), the earmarked fund for CARS‐35, Selection and Breeding of New Local Pig Breeds and Promotion of Industrialization (2024‐XPY‐00‐001), Selection and Breeding of Guangdong small‐ear spotted pig (2022‐440000‐4301030202‐9510), and Chinese Postdoctoral General Program (2025M780258).

## Conflicts of Interest

The authors declare no conflicts of interest.

## Supporting information




**Supporting File**: advs73695‐sup‐0001‐SuppMat.docx.

## Data Availability

All data generated or analyzed during this study are included in this published article, its supplementary information files, and publicly accessible repositories. The RNA‐Seq data for LD muscle samples from GDSS have been deposited in the China National Center for Bioinformation GSA database under accession number GPRJCA054141. Skeletal muscle single‐cell RNA sequencing data utilized in this study are available in the GEO database under accession number GSE138826. Additionally, porcine embryonic muscle single‐cell RNA sequencing data are archived in the China National Center for Bioinformation GSA database under accession number PRJCA036694.
