# Supplementary material for: HMGB2–RAD21 Axis Promotes Fibro/Adipogenic Progenitor Proliferation and Regulates Fat Infiltration
Source: Adv Sci (Weinh). 2026 Jan 12;13(16):e14363. doi: 10.1002/advs.202514363 (PMC13042946; doi:10.1002/advs.202514363)
Supplement: Supplementary file 1 — Supporting File: advs73695‐sup‐0001‐SuppMat.docx. [file ADVS-13-e14363-s001.docx]

Supporting Information

**HMGB2–RAD21 Axis Promotes Fibro/Adipogenic Progenitor Proliferation and Regulates Fat Infiltration**

*Xian Tong;* *Ziyun Liang; Tianqi Duo; Liping Pan; Qi Zhu; Jiete Liang; Xianyao Luo; Qingcai Feng; Rong Xu; Yihao Liu;* *Xu Chen; Luxi Chen;* *Xiaohong Liu*; Yaosheng Chen*; Delin Mo *.*

Xian Tong; Ziyun Liang; Tianqi Duo; Liping Pan; Qi Zhu; Jiete Liang; Xianyao Luo; Qingcai Feng; Rong Xu; Yihao Liu; Xu Chen; Luxi Chen; Xiaohong Liu; Yaosheng Chen; Delin Mo

State Key Laboratory of Biocontrol

School of Life Sciences

Sun Yat-Sen University

Guangzhou 510006, Guangdong, China

*E-mail address:* [modelin@mail.sysu.edu.cn](mailto:modelin@mail.sysu.edu.cn)*;* [chyaosh@mail.sysu.edu.cn](mailto:chyaosh@mail.sysu.edu.cn)*; liuxh8@* *mail.sysu.edu.cn*

Xian Tong, Ziyun Liang, and Tianqi Duo contributed equally to this work.

This PDF file includes:

Supporting text

Tables S1 to S7

Figures S1 to S7

| **Table S1. Antibodies and application used in this study.** | | | | | | |
| --- | --- | --- | --- | --- | --- | --- |
| **Antibody name** | **CN** | **Brand name** | **IF** | **FACS** | **ChIP** | **WB** |
| Anti-PDGFRα(mouse) | 5241S | Cell signaling Technology | 1:200 |  |  |  |
| Anti-PDGFRα(pig) | bs-0231R | bioss | 1:200 |  |  |  |
| Anti-Laminin antibody produced in rabbit | L9393 | Millipore | 1:200 |  |  |  |
| Anti-Perilipin | 9349S | Cell signaling Technology | 1:200 |  |  |  |
| Anti-Ki67 | ab15580 | abcam | 1:200 |  |  |  |
| Anti-HMGB2(HMG2) | H9789 | Millipore | 1:200 |  | 1:50 | 1:1000 |
| Anti-IgG | #2729S | Cell signaling Technology |  |  | 1:50 |  |
| Anti-rat IgG(H+L) Alexa Fluor(R) 488 | #4416S | Cell signaling Technology | 1:1000 |  |  |  |
| PE anti-mouse CD140a Antibody | 135906 | Biolegend |  | 0.25 µg / million cells |  |  |
| FITC anti-CD45 | 157214 | Biolegend |  | 0.25 µg / million cells |  |  |
| APC anti-mouse CD31 Antibody | 102410 | Biolegend |  | 0.25 µg / million cells |  |  |

Note: CN: Catalogue Number; IF: Immunofluorescence; WB: Western Blot.

| **Table S2. List of primers used in this study.** | | | |
| --- | --- | --- | --- |
| **Gene** | | **Forward 5’-3’** | **Reverse 5’-3’** |
| **RT-pcr** | | | |
| HMGB2 | AAGAGCGACAAAGCTCGTTATG | | GCAGTATCTCCAATAGACAGGC |
| Cyclin D1 | CAGCAGGAGCTAAAGCCGAA | | TCGTTGAGGAGGTTGGCATC |
| Cyclin E2 | ATGTCAAGACGCAGCCGTTTA | | GCTGATTCCTCCAGACAGTACA |
| CDK2 | GCTCTCCTTGCGTTCCATCC | | ACGTGCCCTCTCCAATCTTC |
| RAD21 | GGATAAGAAGCTAACCAAAGCCC | | CTCCCAGTAAGAGATGTCCTGAT |
| β-actin | ACGGCCAGGTCATCACTATTG | | TGGATGCCACAGGATTCCA |
| **siRNA-sequence** | | | |
| NC | UUCUCCGAACGUGUCACGUTT | | ACGUGACACGUUCGGAGAATT |
| HMGB2 | GCCUGUCUAUUGGAGAUACUGCGAA | | UUCGCAGUAUCUCCAAUAGACAGGC |
| **ChIP-qPCR** | | | |
| RAD21 | TCCCAAAAATCTTGTCAATCCTCT | | TCCTCGGAACTGATCAACTCTTG |

| **Table S3 sgRNA for generating HMGB2^-/-^ mice (5’ to 3’)** | | |
| --- | --- | --- |
| **Names** | **Sequence** | **PAM** |
| 5'Guide | CGCGCCCCCGCGAGCGAGCG | CGG |
| 3'Guide | AGGTCTTAAGTCCTAGGTAG | AGG |

| **Table S4 Primers for genotyping of HMGB2^-/-^ mice (5’ to 3’)** | | | |
| --- | --- | --- | --- |
| **NO.** | **Name** | **Sequence** | **Product size** |
| 1 | HMGB2-WT-F | GGTCCCAGAATGAACTGAGGGCTTC | WT: 387bp |
|  | HMGB2-WT-R | CCGCTGGGAATCTCAGGGAAAACTA |  |
| 2 | HMGB2-Mut-F | TCGCGGTGGCTTGTGTAAGTGTAAG | Mut: 431bp |
|  | HMGB2-WT-R | CCGCTGGGAATCTCAGGGAAAACTA |  |

| **Table. S5 Summary of the basic data of RNA-seq library** | | | | | | | | |
| --- | --- | --- | --- | --- | --- | --- | --- | --- |
| **Name** | **RawReads** | **RawBases** | **CleanReads** | **CleanBases** | **CleanRatio** | **Q20** | **Q30** | **GC%** |
| E35_1 | 45265628 | 6789844200 | 44565968 | 6.53E+09 | 98.45% | 97.56% | 93.41% | 57.28% |
| E35_2 | 47335110 | 7100266500 | 46588830 | 6.798E+09 | 98.42% | 97.38% | 93.05% | 56.09% |
| E35_3 | 48028574 | 7204286100 | 47331058 | 6.93E+09 | 98.55% | 97.33% | 92.90% | 56.24% |
| E50_1 | 46646878 | 6997031700 | 45751568 | 6.638E+09 | 98.08% | 97.30% | 92.86% | 55.98% |
| E50_2 | 50191876 | 7528781400 | 49070378 | 7.168E+09 | 97.77% | 97.11% | 92.47% | 56.27% |
| E50_3 | 50481660 | 7572249000 | 49341914 | 7.172E+09 | 97.74% | 97.28% | 92.86% | 56.17% |
| E73_1 | 45858552 | 6878782800 | 45335864 | 6.667E+09 | 98.86% | 97.48% | 93.18% | 54.33% |
| E73_2 | 52076584 | 7811487600 | 51509972 | 7.539E+09 | 98.91% | 97.43% | 93.09% | 54.84% |
| E73_3 | 50374556 | 7556183400 | 49823802 | 7.29E+09 | 98.91% | 97.40% | 93.05% | 54.94% |
| E99_1 | 47683296 | 7152494400 | 47154226 | 6.94E+09 | 98.89% | 97.39% | 93.01% | 55.18% |
| E99_2 | 53490922 | 8023638300 | 52873390 | 7.713E+09 | 98.85% | 97.55% | 93.38% | 56.12% |
| E99_3 | 50497820 | 7574673000 | 49919374 | 7.297E+09 | 98.85% | 97.51% | 93.30% | 55.96% |
| P2_1 | 37880908 | 5682136200 | 37561312 | 5.604E+09 | 99.16% | 97.68% | 93.34% | 51.30% |
| P2_2 | 38516598 | 5777489700 | 38063194 | 5.674E+09 | 98.82% | 97.70% | 93.52% | 51.03% |
| P2_3 | 39566066 | 5934909900 | 39035980 | 5.82E+09 | 98.66% | 97.52% | 93.15% | 55.40% |
| P9_1 | 37444376 | 5616656400 | 36855248 | 5.493E+09 | 98.43% | 97.80% | 93.77% | 58.53% |
| P9_2 | 42362270 | 6354340500 | 41905484 | 6.256E+09 | 98.92% | 97.79% | 93.69% | 58.37% |
| P9_3 | 53039432 | 7955914800 | 52540944 | 7.841E+09 | 99.06% | 97.73% | 93.50% | 58.21% |
| P80_1 | 42074828 | 6311224200 | 41659564 | 6.217E+09 | 99.01% | 98.00% | 94.22% | 58.31% |
| P80_2 | 42464050 | 6369607500 | 42137366 | 6.292E+09 | 99.23% | 97.93% | 93.93% | 56.30% |
| P80_3 | 51377602 | 7706640300 | 50984864 | 7.611E+09 | 99.24% | 97.89% | 93.89% | 56.78% |
| P180_1 | 41398558 | 6209783700 | 41058546 | 6.128E+09 | 99.18% | 97.81% | 93.68% | 54.48% |
| P180_2 | 49488428 | 7423264200 | 49133112 | 7.335E+09 | 99.28% | 97.92% | 93.93% | 55.79% |
| P180_3 | 40072596 | 6010889400 | 39699054 | 5.926E+09 | 99.07% | 97.90% | 93.96% | 57.06% |

| **Table S6 Alignment of Transcriptome Sequences with Reference Sequences** | | | | | |
| --- | --- | --- | --- | --- | --- |
| **Sample** | **Total Reads** | **Mapped Reads** | **Map Rate** | **unique mapped Reads** | **unipue map Rate** |
| E35_1 | 44565968 | 42357384 | 95.04% | 40403631 | 90.66027916 |
| E35_2 | 46588830 | 44110412 | 94.68% | 42176394 | 90.52898302 |
| E35_3 | 47331058 | 44992360 | 95.06% | 42237261 | 89.23793971 |
| E50_1 | 45751568 | 43241554 | 94.52% | 41213450 | 90.08095635 |
| E50_2 | 49070378 | 46403828 | 94.57% | 42486740 | 86.58327433 |
| E50_3 | 49341914 | 46642349 | 94.53% | 41943529 | 85.00588161 |
| E73_1 | 45335864 | 43235581 | 95.37% | 39662547 | 87.48602872 |
| E73_2 | 51509972 | 48803246 | 94.75% | 43262878 | 83.98932541 |
| E73_3 | 49823802 | 47246137 | 94.83% | 44434399 | 89.18307559 |
| E99_1 | 47154226 | 44638765 | 94.67% | 42544722 | 90.22465981 |
| E99_2 | 52873390 | 50099540 | 94.75% | 47610689 | 90.04659811 |
| E99_3 | 49919374 | 47226771 | 94.61% | 44583653 | 89.31132229 |
| P2_1 | 37561312 | 36225956 | 96.44% | 35074779 | 93.38006883 |
| P2_2 | 38063194 | 36354264 | 95.51% | 34960003 | 91.84726589 |
| P2_3 | 39035980 | 37372502 | 95.74% | 35351108 | 90.56031897 |
| P9_1 | 36855248 | 34995018 | 94.95% | 33341313 | 90.46557766 |
| P9_2 | 41905484 | 40406660 | 96.42% | 38222232 | 91.21057282 |
| P9_3 | 52540944 | 50764747 | 96.62% | 47754455 | 90.88998287 |
| P80_1 | 41659564 | 40193802 | 96.48% | 38332753 | 92.01429232 |
| P80_2 | 42137366 | 40811274 | 96.85% | 38923057 | 92.37183216 |
| P80_3 | 50984864 | 49407299 | 96.91% | 47046853 | 92.27611748 |
| P180_1 | 41058546 | 39503338 | 96.21% | 37892520 | 92.2889963 |
| P180_2 | 49133112 | 47576328 | 96.83% | 45470548 | 92.54562992 |
| P180_3 | 39699054 | 38176122 | 96.16% | 36351950 | 91.56880665 |

**Table S7. The top10 differentially expressed genes in different FAPs subpopulations**

| **Gene** | **pct.HMGB2+ Fibroblast** | **pct.HMGB2- Fibroblast** | **avgExp.HMGB2+ Fibroblast** | **avgExp.HMGB2- Fibroblast** | **avg_logFC.HMGB2+ Fibroblast_vs_HMGB2- Fibroblast** | **p_val** | | **p_val adj** |
| --- | --- | --- | --- | --- | --- | --- | --- | --- |
| TOP2A | 0.72 | 0.052 | 9.206581 | 0.369814 | 2.008358 | 0 | 0 | |
| CENPF | 0.64 | 0.034 | 7.389836 | 0.24796 | 1.905511 | 0 | 0 | |
| HMGB2 | 0.936 | 0.25 | 14.49582 | 1.451801 | 1.843748 | 0 | 0 | |
| Ki67 | 0.775 | 0.082 | 7.98271 | 0.439659 | 1.830896 | 0 | 0 | |
| TPX2 | 0.676 | 0.037 | 4.611398 | 0.185807 | 1.554376 | 0 | 0 | |
| CENPE | 0.6 | 0.021 | 3.953567 | 0.110701 | 1.495117 | 0 | 0 | |
| UBE2C | 0.553 | 0.029 | 4.112167 | 0.178251 | 1.467592 | 0 | 0 | |
| ENSSSCG00000017032 | 0.558 | 0.028 | 3.79194 | 0.130742 | 1.444061 | 0 | 0 | |
| PCLAF | 0.567 | 0.033 | 3.620655 | 0.200735 | 1.347603 | 0 | 0 | |
| DUT | 0.862 | 0.197 | 6.468535 | 0.9995 | 1.317802 | 0 | 0 | |
| H2AFV | 0.932 | 0.441 | 10.9527 | 2.313885 | 1.282836 | 0 | 0 | |


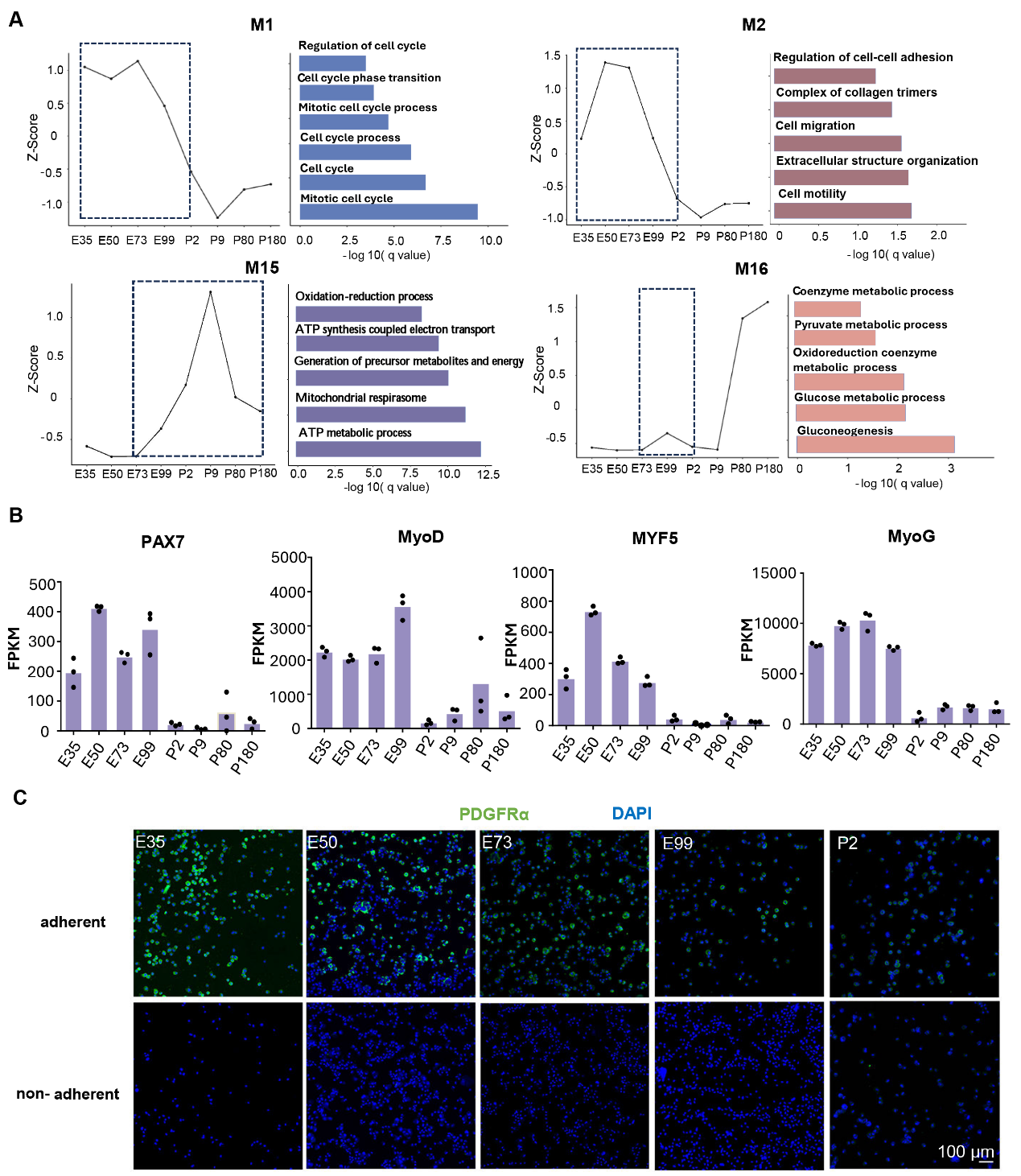


Figure S1. Gene co-expression network analyses during skeletal muscle development

(A) Expression Changes and GO Enrichment Analysis of Modules 1,2, 15 and 16. The left panel shows the median Z score of genes in each module across skeletal muscle development, while the right panel lists the top five typical GO biological process terms associated with each module. (B) Changes in FPKM levels of Myogenesis genes (PAX7, Myod, MYF5, and MyoG) during skeletal muscle development. (D) Validation of FAPs Extraction from Various Samples.


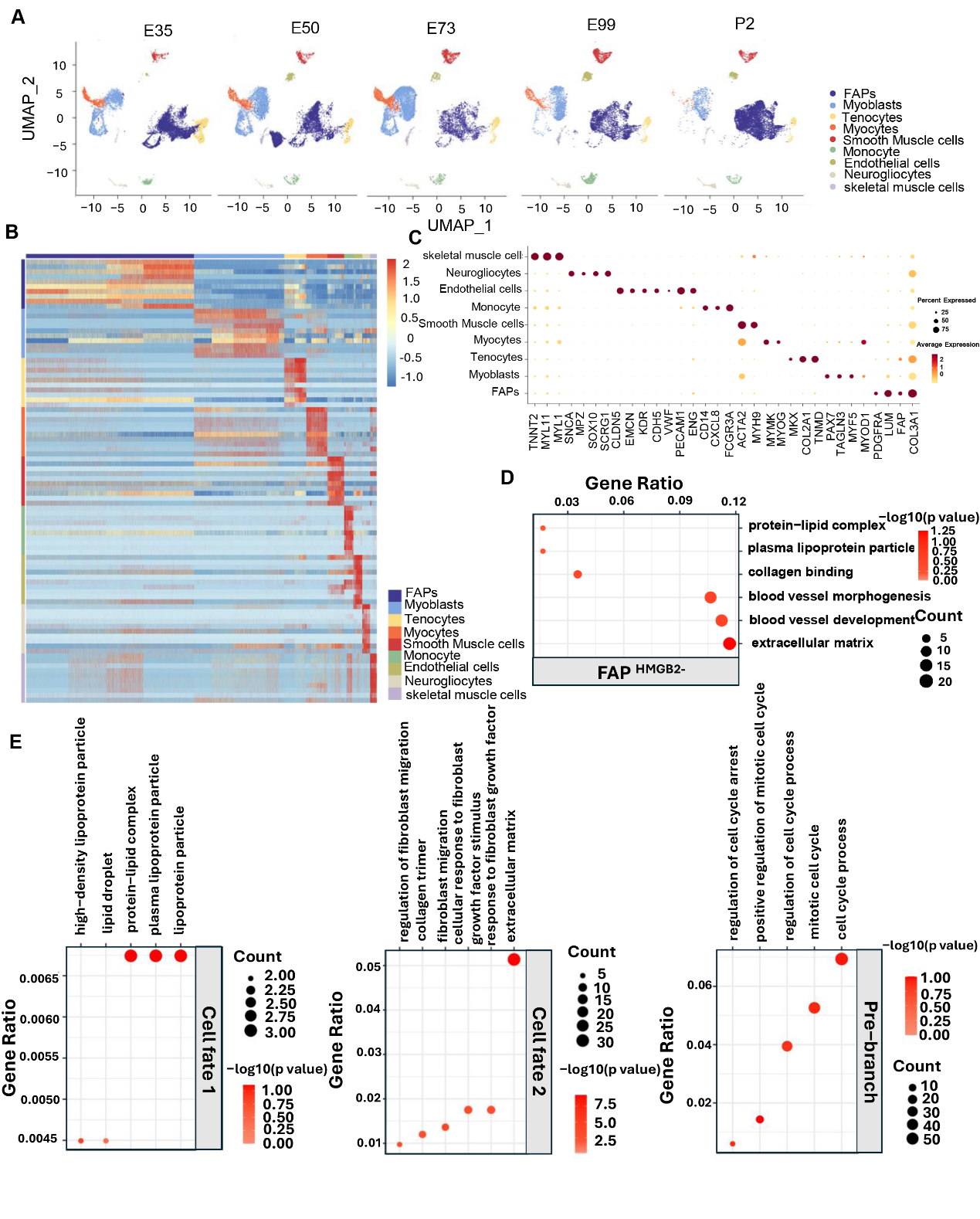


Figure S2. Single-cell RNA Sequencing analysis of Skeletal Muscle During Embryonic Development

(A) Graph-based clustering of the isolated single cells reveals distinct clusters representing different cell populations. (B) Heatmap depicting the top 10 differentially expressed genes in each cell cluster. (C) Bubble plot illustrating the specific highly expressed genes in each cell cluster. (D) GO enrichment analysis of FAP^HMGB2-^ cluster. (E) GO enrichment analysis of three cell states (Pre-branch, Cell fate 1, and Cell fate 2).


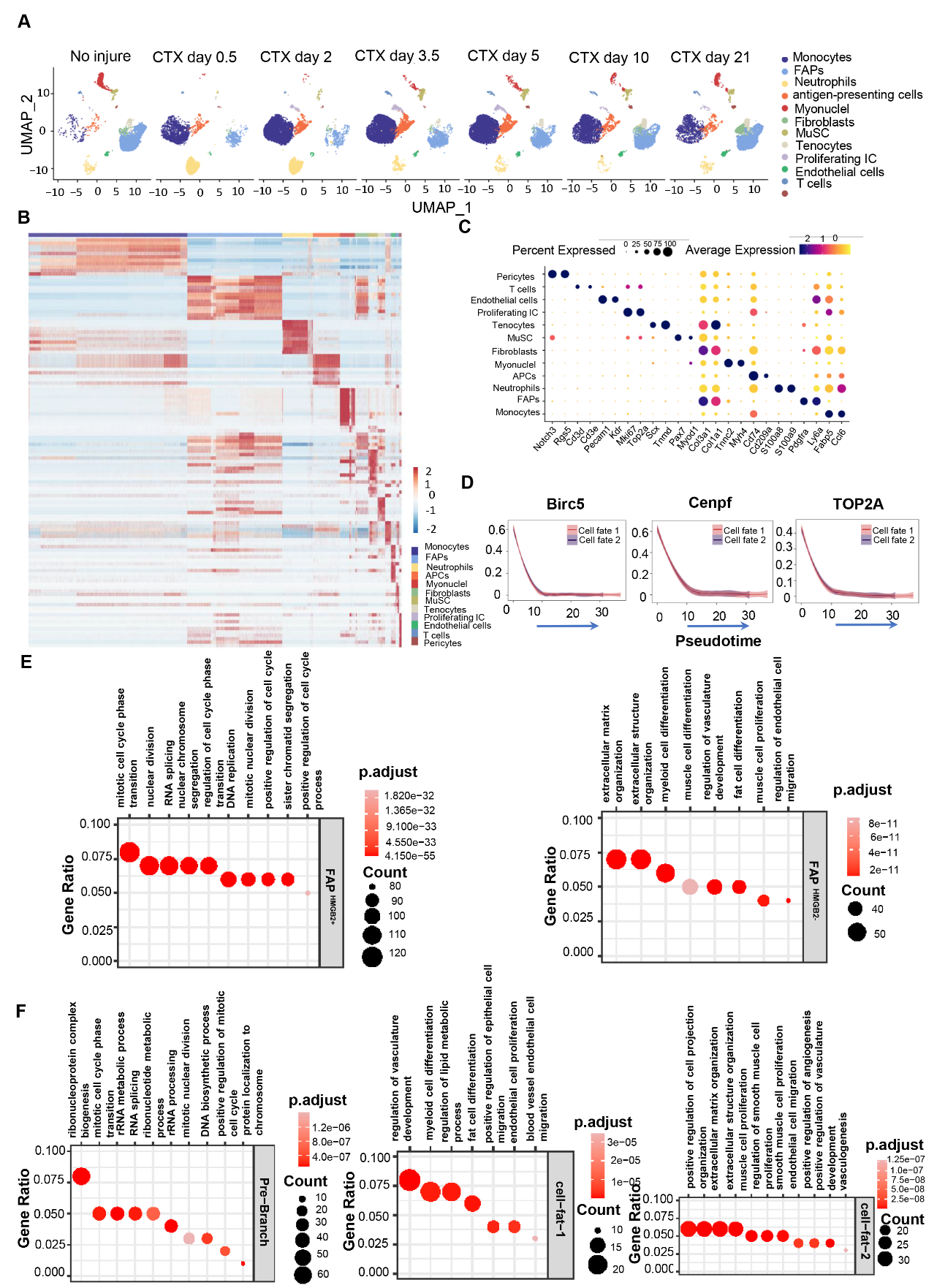


Figure S3. Single-cell RNA Sequencing analysis of Skeletal Muscle During Muscle Injury

(A) Graph-based clustering of the isolated single cells reveals distinct clusters representing different cell populations. (B) Heatmap depicting the top 10 differentially expressed genes in each cell cluster. (C) Bubble plot illustrating the specific highly expressed genes in each cell cluster. (D) Dynamic Expression of Proliferation-Associated Genes (such as Ki67, CENPF, and Top2A). (E) GO enrichment analysis of FAP^HMGB2-^ cluster and FAP pro cluster. (F) GO enrichment analysis of three cell states (Pre-branch, Cell fate 1, and Cell fate 2).


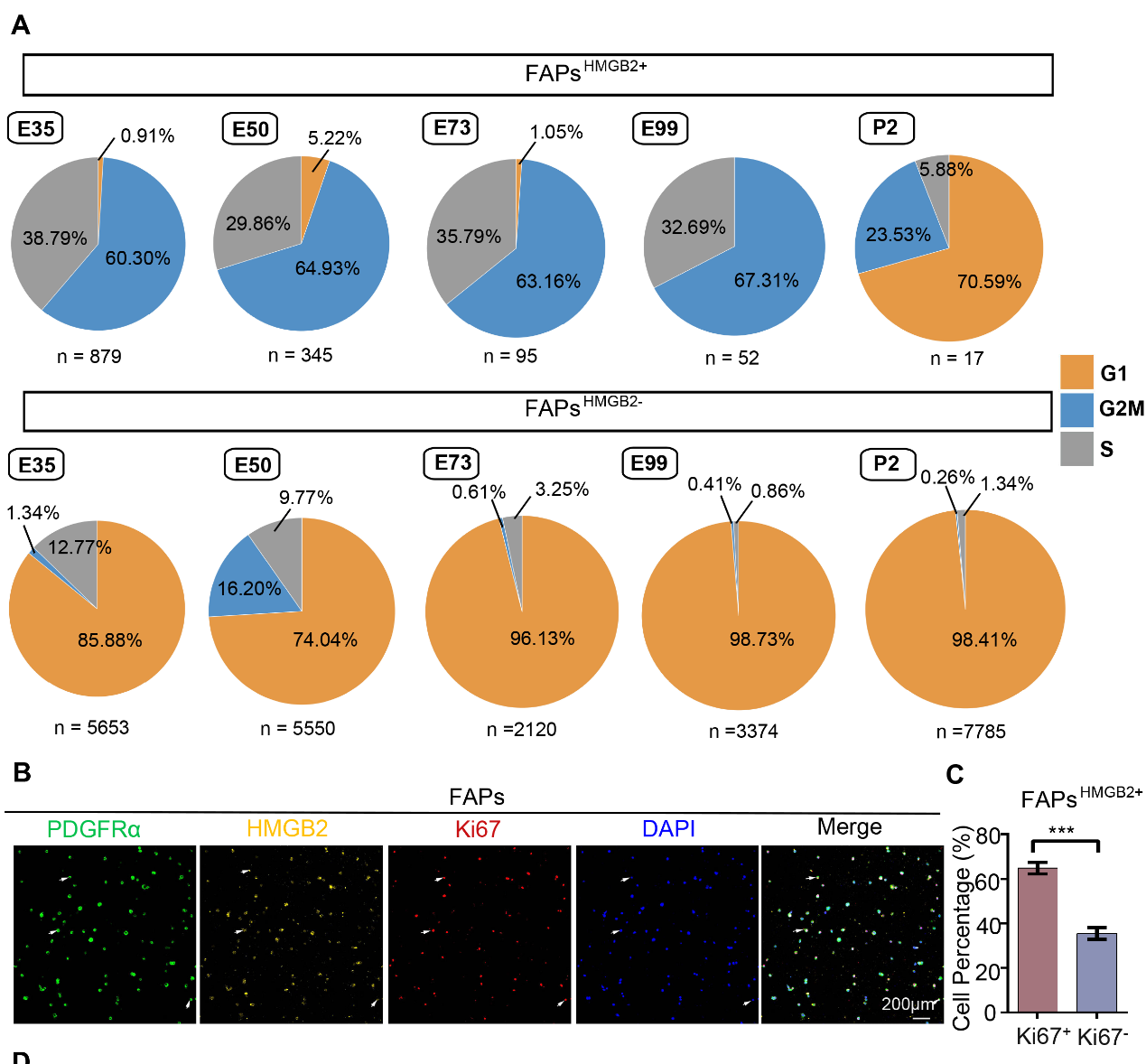


**Figure S4. Cell cycle statistics of FAPs^HMGB2+^ cell clusters**

(A) Exploring cell cycle analysis of FAPs subpopulations at different developmental stages based on single-cell transcriptome data; (B) Multiplex immunofluorescence staining was performed for PDGFRα, HMGB2, and Ki67 in FAPs; (C) The proportion of Ki67 positive cells was quantified within the FAPs^HMGB2⁺^ cell population in FAPs.


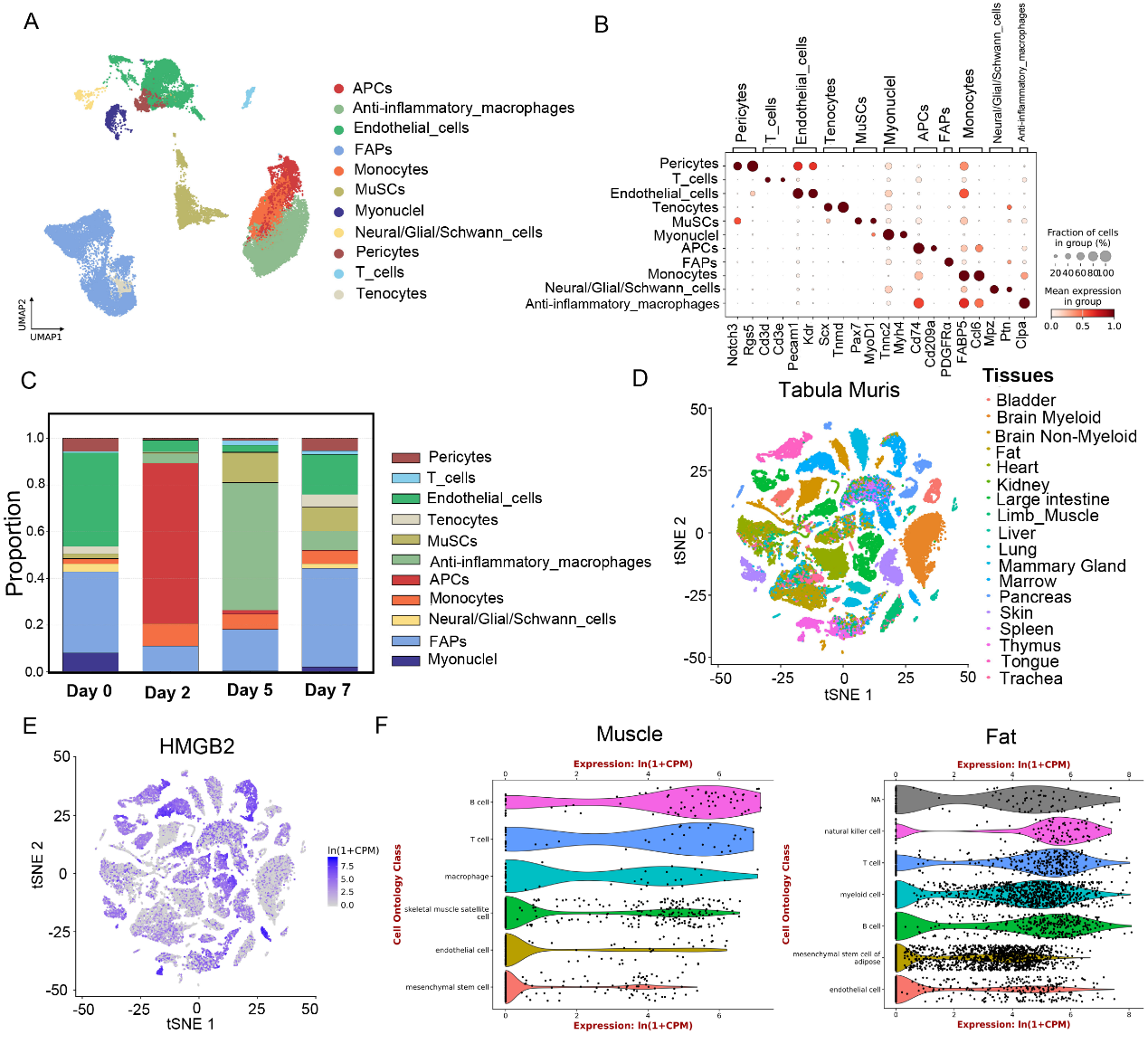


**Figure S5. Exploration of each part of the results based on public databases**

(A-C) Cell clustering (A), bubble plot generation (B), and cell proportion analysis (C) during muscle injury were performed using public data (GEO: GSE143437). (D-E) HMGB2 expression across various organ and tissue types was analyzed using the Tabula Muris database. (F) HMGB2 expression levels across different cell types in muscle and adipose tissues.


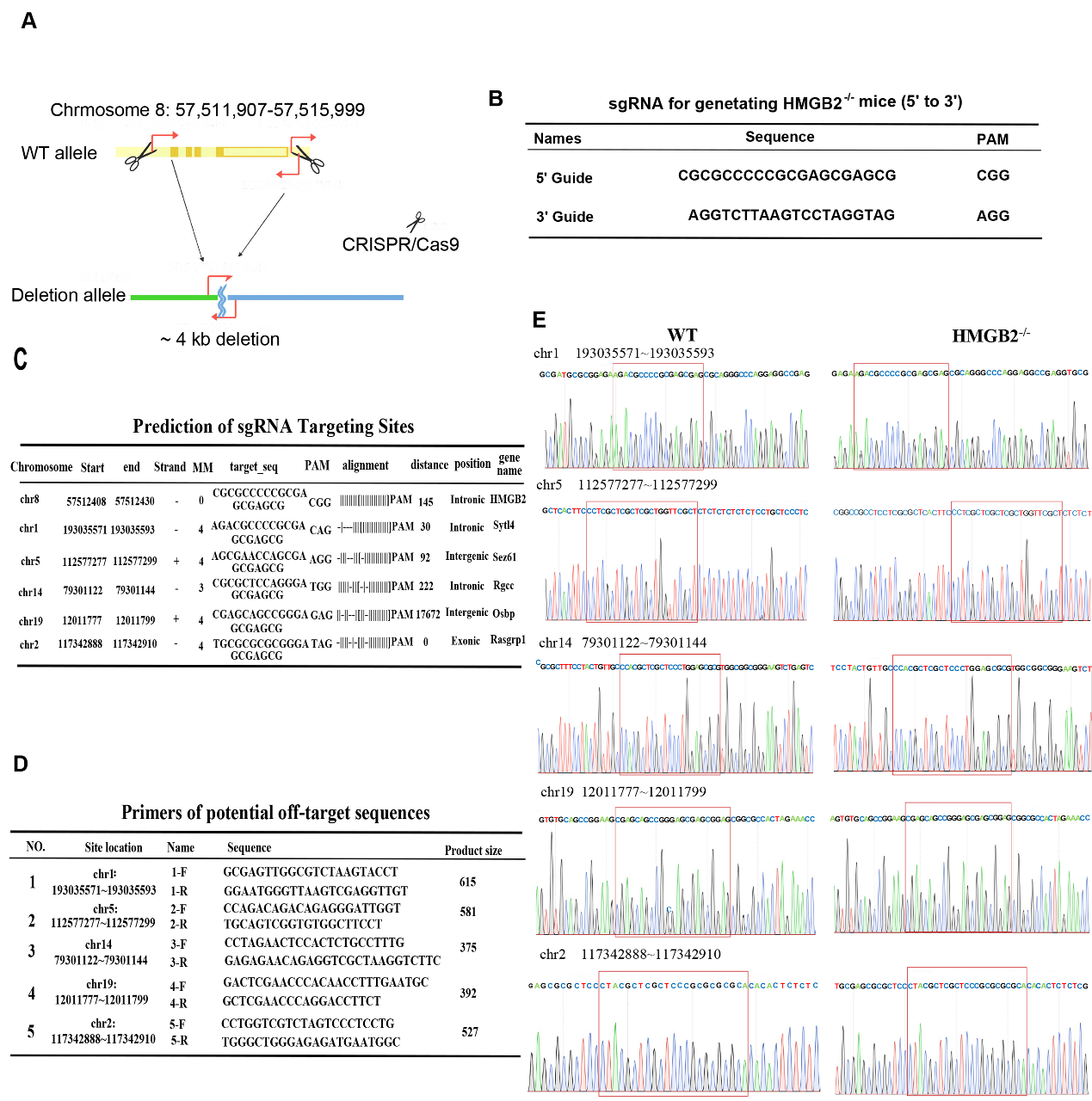


**Figure S6. Construction of HMGB2 knockout mice**

(A) Design and confirmation of hmgb2-/- mice. (B) sgRNA for generating HMGB2-/- mice (5’ to 3’). (C) Predict the targeted regions of sgRNA; (D) Primer design for potential off-target genes. (E) The top five predicted off-target sites were validated by Sanger sequencing based on PCR amplification of each candidate locus.

**
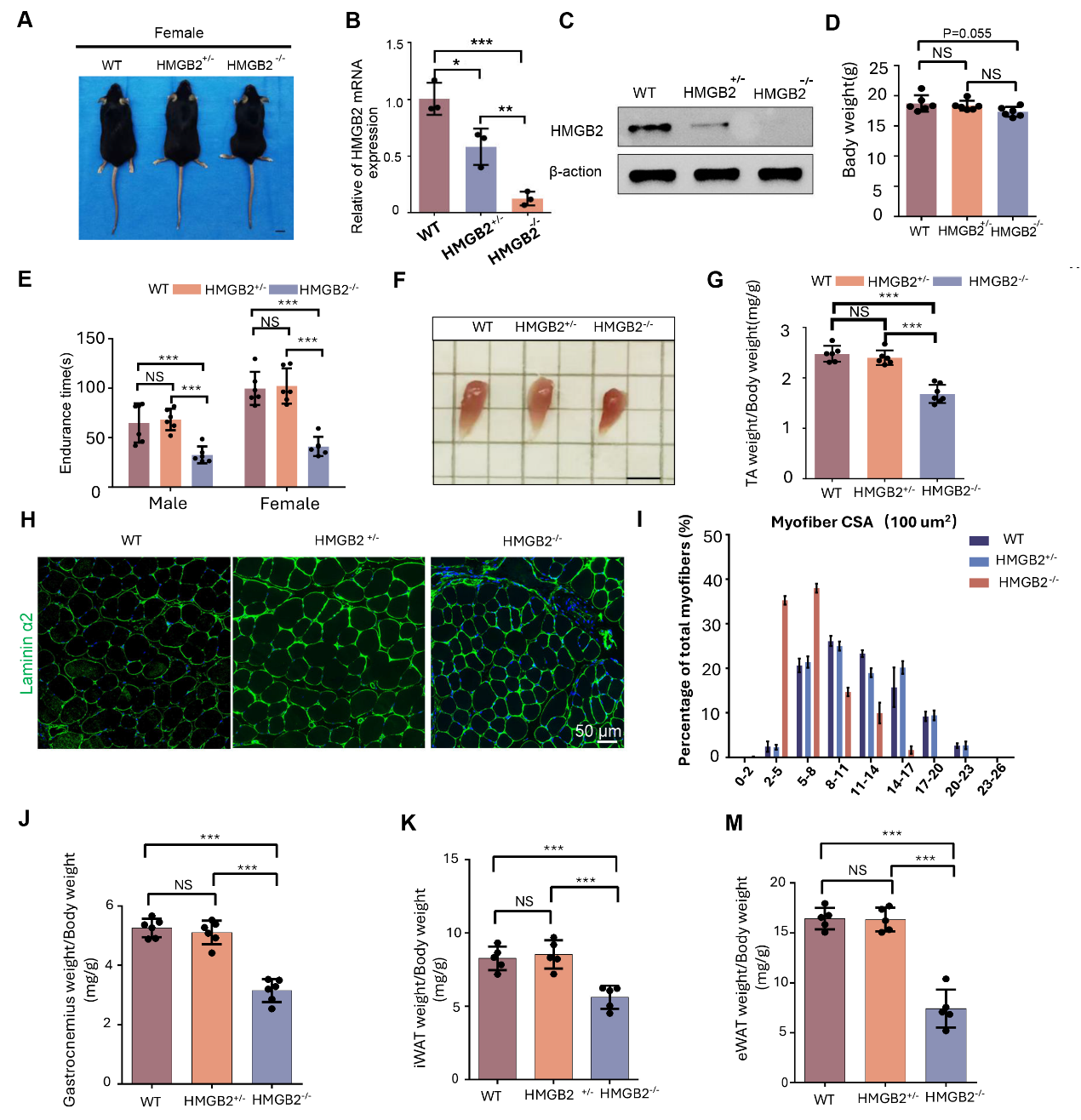
**

Figure S7. Effects of HMGB2 knockdown on muscle development

(A) Representative images of 8-week-aged control HMGB2^+/-^and HMGB2^-/-^ female mice. Scale bar = 1 cm. (B) qPCR analysis of HMGB2 expression. (C) Western blot analysis of HMGB2 expression. (D)The body weight of mice with different genotypes at 8 weeks of age (n = 6 for each genotype); (E) Assessment of skeletal muscle strength through grip strength experiment (n = 6 for each genotype); (F) Representative images of tibialis anterior (TA) muscles in male mice of various genotypes at 8 weeks of age, scale = 0.5 cm; (G) Quantification of TA weight/body weight; (H) Immunofluorescence staining of Laminin α2 was performed on cross sections of TA of each genotype at 8 week; (I) Distribution of cross-sectional area of muscle fibers for each genotype. (J) Quantification of Gastrocnemius weight/body weight for each genotype. (K) Quantification of iWAT weight/body weight for each genotype. (M) Quantification of eWAT weight/body weight for each genotype.
